# 2018 Survey of antimicrobial drug use and stewardship practices in adult cows on California dairies: post-Senate Bill 27

**DOI:** 10.7717/peerj.11515

**Published:** 2021-07-13

**Authors:** Pius S. Ekong, Essam M. Abdelfattah, Emmanuel Okello, Deniece R. Williams, Terry W. Lehenbauer, Betsy M. Karle, Joan D. Rowe, Edith S. Marshall, Sharif S. Aly

**Affiliations:** 1 Veterinary Medicine Teaching and Research Center, School of Veterinary Medicine, University of California, Davis, Tulare, CA, United States; 2 Department of Epidemiology, National Veterinary Research Institute, Vom, Plateau, Nigeria; 3 Department of Animal Hygiene, and Veterinary Management, Faculty of Veterinary Medicine, Benha University, Qalubiya Governorate, Egypt; 4 School of Veterinary Medicine, Department of Population Health and Reproduction, University of California, Davis, Davis, CA, United States; 5 Cooperative Extension, Division of Agriculture and Natural Resources, University of California, Orland, CA, United States; 6 Antimicrobial Use and Stewardship, Animal Health and Food Safety Services Division, California Department of Food and Agriculture, Sacramento, CA, United States

**Keywords:** California dairy industry survey, Antimicrobial resistance, Antimicrobial stewardship, Antimicrobial drugs (AMD), Judicious use of AMD

## Abstract

**Background:**

A survey of California (CA) dairies was performed in spring 2018 to characterize antimicrobial stewardship practices, antimicrobial drug (AMD) use, and health management of adult cows on CA dairies since the implementation of the Veterinary Feed Directive (VFD) and the CA Senate Bill 27 (SB 27). Effective January 1, 2017, the U.S. Food and Drug Administration (FDA) implemented regulatory changes requiring veterinary oversight for therapeutic uses of medically-important antimicrobial drugs (MIADs) administered in feed (VFD) and water (veterinary prescription). Similarly, effective January 1, 2018, the CA legislature enacted California Food and Agricultural Code (FAC) 14400–14408, formerly known as Senate Bill 27 (SB 27) requiring veterinary prescriptions for all other dosage forms of MIADs.

**Methods:**

The questionnaire consisted of 43 questions partitioned into three sections to assess herd information, management practices, and AMD use and perspectives. The questionnaire was mailed to 1,282 grade A licensed dairies in CA and 149 responses (11.6%) were collected from 19 counties across the three defined regions of CA: Northern CA (NCA), Northern San Joaquin Valley (NSJV), and Greater Southern CA (GSCA).

**Results:**

Most dairies reported treating all dry cows with intramammary AMD and/or teat sealant at the end of a lactation (87.2%). In 92.3% of dairies, producers relied on the veterinarian for information about AMD used to treat cows. Treatment duration for cows treated with AMD was based on the drug manufacturer’s label and veterinarian’s instructions in most dairies (98.6%). Most respondents to the survey confirmed having a valid veterinarian-client-patient-relationship (VCPR) for their dairies (91.7%), participated in animal welfare audit programs (81.8%) and dairy quality assurance programs (52.9%). Approximately 98.6% respondents were aware that all uses of MIADs in livestock required a veterinary feed directive (VFD) or prescription and are no longer sold over-the-counter (OTC) in CA since January 1, 2018. Multiple factor analysis (MFA) was performed and identified seven components composed of 21 variables (questions) that explained 99.7% of the total variance in the data. Hierarchical cluster analysis on the principal coordinates of the MFA based on conventional dairy survey responses identified two clusters characterized as large conventional dairies (median herd size: 1,265 cows) and mid-sized conventional dairies (median herd size: 715 cows) mostly in GSCA and NSJV. The organic dairies grouped into a single cluster of median herd size of 325 cows mostly in NCA.

**Conclusions:**

The survey results contribute to the knowledge of AMD use and antimicrobial stewardship practices on CA dairies since the implementation of the SB 27 and VFD laws and provide useful information for future evaluation of resistance-related risk in adult cows.

## Introduction

California (CA) is the leading dairy producing state in the United States, with over 1.7 million dairy cows producing 18.5 percent of the nation’s milk supply on 1,331 dairies (California Department of Food and Agriculture (CDFA), 2018). Maintaining the health and welfare of the cattle population is crucial for the continued productivity of CA’s dairy industry. While good management practices are key to maintaining dairy cattle health, the continued efficacy of antimicrobial drugs (AMD) is required to safeguard our herds’ health and welfare. However, with the misuse and overuse of AMDs in human medicine and food production comes the risk of antimicrobial resistance (AMR) affecting both animal and human populations. As a result, the continued efficacy of AMD against bacterial diseases is essential for public and animal health. According to the World Health Organization, AMR poses a great threat to modern medicine and the sustainability of an effective, global public health response to the enduring threat from infectious diseases (World Health Organization (WHO), 2015). According to the U.S. Center for Disease Control and Prevention (CDC), more than 2.8 million antibiotic-resistant infections occur in the U.S. each year, resulting in more than 35,000 human deaths and considerable costs to the U.S. healthcare system (U.S. Centers for Disease Control and Prevention (CDC), 2019). However, the effect that antibiotic use in food-producing animals has on the occurrence of AMR infection in humans remains unclear (Tang et al., 2017).

The development of AMR is a complex multifactorial process driven by numerous factors (Allen et al., 2010; Benedict et al., 2015). Exposure to AMD by people, animals, or the environment (crops, soil, water) plays a large role in this development of AMR (Aarestrup, 2015; Jayarao, Almeida & Oliver, 2019; Levy & Marshall, 2004; Witte, 2000; Wright, 2010). However, good antimicrobial stewardship, which includes judicious AMD use is necessary to: ensure the continued availability of antimicrobials effective against infections in people and animals; guarantee the health of dairy herds; ensure a continuous supply of wholesome milk and dairy beef; and protect public health.

Understanding the complex pathways between on-farm AMD use and AMR in the dairy cattle population is key to antimicrobial stewardship and judicious use. In response to the growing concern surrounding AMR, the U.S. Food and Drug Administration (FDA) in June 2015 announced stricter requirements for administering medically important antimicrobial drugs (MIADs) to animals through feed and water. The law, known as the Veterinary Feed Directive (VFD), mandates supervision by a licensed veterinarian for any use of MIADs in animal feed as of January 1^st^ 2017 (U.S Food and Drug Administration (FDA), 2015). California Senate Bill 27 (SB 27; Hill) was passed in late 2015 and resulted in the Livestock: Use of Antimicrobial Drugs Law (California Food and Agricultural Code, FAC Sections 14400–14408) (Food and Agricultural Code (FAC), 2015). The FAC law, here onwards referred to as SB 27, removed all remaining MIADs from over-the-counter (OTC) status and requires veterinary oversight and prescription for their administration in livestock, effective January 1^st^ 2018 (California State Senate, 2015; California Department of Food and Agriculture (CDFA), 2019). The law also created the Antimicrobial Use and Stewardship (AUS) program within the California Department of Food and Agriculture (CDFA) and mandates that, in consultation with the Veterinary Medical Board, the State Department of Public Health, universities, and cooperative extensions the program develop antimicrobial stewardship guidelines and best management practices on the proper use of MIADs. Senate Bill 27 also requires CDFA to gather information on MIAD sales and usage, AMR bacteria, and livestock management practice data. At the time our study was initiated, it was unknown what impact the implementation of the VFD and SB 27 have had on management and/or AMD use on CA dairies. The objective of the survey was to characterize antimicrobial stewardship practices, AMD use, and health management of adult cows on CA dairies since the implementation of the VFD and SB 27. The associations between the management practices on conventional dairies and key antimicrobial stewardship and health outcomes are described elsewhere (Ekong et al., 2021).

## Materials and Methods

### Questionnaire design

A survey instrument was designed to collect information about AMD use in adult cows on California dairies. The questionnaire was pre-tested using in-person interviews with extension and outreach specialists and veterinarians. The questionnaire consisted of 43 questions partitioned into three sections: herd information, health management, AMD use, practices and perspectives. Of the 43 questions, 40 were multiple choice questions with the option to specify any available additional information, three questions required the respondent to fill in the requested information. An optional section was included to allow respondents to provide contact information if they were interested in participating in a continuation study and to provide feedback about the questionnaire. The study was reviewed by the University of California, Davis Institutional Review Board and was granted exemption approval (IRB Number: 1537295-1).

### Questionnaire administration and data collection

A list of all 1,282 licensed, grade A dairies in CA in 2017 provided by CDFA served as a sampling frame. A grade A dairy produces milk used in any products intended for consumption in fluid form (also called fluid grade milk or market milk). A confidential identification number was randomly assigned to all dairies on the list. Each dairy was uniquely identified by this number during analysis to maintain confidentiality. Dairies were mailed a survey packet containing a copy of the questionnaire, a postage-paid addressed business reply envelope, and an information cover letter. The cover letter introduced the dairy producers to the survey and provided an explanation that the term antimicrobial included antibiotics, drugs that are naturally produced by other microorganisms and that can kill or inhibit the growth of other microorganisms, and also synthetic chemicals such as sulfonamides. Producers were instructed that for the purpose of this survey questions will refer to all AMDs as antibiotics regardless of their origin. The estimated time for producers to complete the survey was 30 minutes. To improve questionnaire response rate, each dairy was mailed a reminder card two weeks after the initial questionnaire package. A second and third copy of the questionnaire, each followed by a reminder card, were similarly mailed a month and six months later, respectively, to dairies that had not previously responded. All mailings were completed between April and September 2018.

### Questionnaire sections

#### Herd information

This section focused on gathering baseline information about the responding dairy. Questions included: the respondent’s role on the dairy, the county where the dairy was located, the breeds of milking cows, the number of milking cows (herd size), the annual rolling herd average for milk production (RHA), the previous month’s average bulk tank somatic cell count in the herd (BTSCC), and if the dairy was a USDA certified organic milk producer.

#### Dairy cow health management and antimicrobial use

The questions in this section addressed the farms’ dry-off protocols, types of AMD used at dry-off, disease prevention, and vaccinations used in lactating cows. Additional questions highlighted sources of information on AMD used to treat sick cows, who made the decisions on what AMD are stocked or used to treat sick cows, and whether producers had written or computerized animal health protocols or used a drug inventory log on their dairies. Other questions were considered indicators for AMD usage on the dairy, such as how and which AMD treatment information is tracked, if the dairy tracked AMD withdrawal intervals for treated cows, the farm’s disease diagnosis and management practices, and if the dairy had a working relationship with a veterinarian or veterinary practice.

#### Practices and perspectives

The final section posed questions relating to the respondent’s participation in animal welfare audit programs and/or dairy quality assurance programs, the respondent’s familiarity with the FDA’s “medically important antimicrobial drugs (MIADs)” term, and awareness that all MIADs required a prescription and were no longer sold OTC in CA after January 1, 2018. Additional questions addressed differences that may have occurred in the periods before and after January 1, 2018 in: the use of OTC and prescription AMD on the dairy, changes made regarding previously available OTC AMD, and changes in the dairy’s AMD drug costs. Similarly, questions inquired about differences before and after January 1, 2018, in usage of alternatives to AMD (defined as vaccines, vitamins, minerals, herbal remedies or others), any changes in management to prevent a disease outbreak or disease spread, and changes noted in animal health on the dairy. The final questions in this section considered how important survey respondents ranked certain AMD use stewardship practices on the dairy, and their level of agreement on statements relating to AMR.

### Statistical analysis

#### Exploratory data analysis

**Figure 1:**
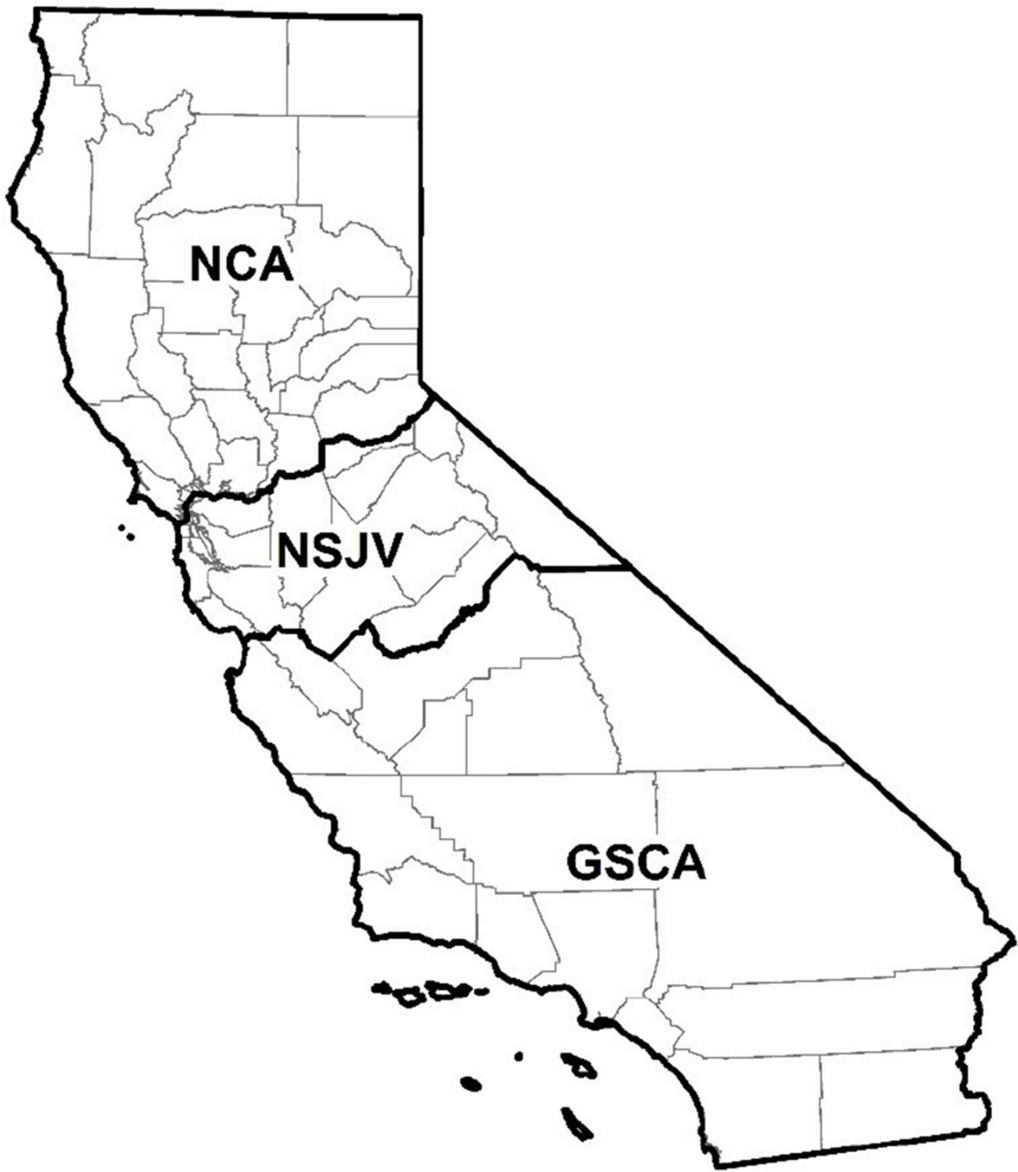
Map of counties in northern California (NCA), northern San Joaquin Valley (NSJV), and greater southern California (GSCA) regions for comparison of antimicrobial drug use and stewardship practices.

Data on the counties where the dairies were located were categorized into three regions (Fig. 1)—Northern California (NCA), Northern San Joaquin Valley (NSJV), and Greater Southern California (GSCA) (Love et al., 2016). For the purposes of descriptive analysis, the herd size and RHA on respondent dairies were categorized based on statewide herd distribution. The statewide mean herd size and RHA was 1,304 milking cows and 23,500 lbs/cow, respectively (California Department of Food and Agriculture (CDFA), 2018). We categorized herd size as <1,305 milking cows (less than the state mean), 1,305 to 3,500 milking cows (larger herds), and >3,500 milking cows to distinguish the largest herds in the state. We categorized RHA as <10,660 kg/cow (<23,500 lbs/cow), less than the state mean; and ≥10,660 kg/cow. BTSCC was categorized as <100,000, 100,000–199,999, and ≥200,000 cells/mL. The <100,000 cells/mL BTSCC represent the high quality milk herd designation commonly associated with bonus payment to such dairies. The 100,000–199,999 cells/mL category represent the industrywide acceptable goal for BTSCC in dairy herds, while the category with ≥200,000 cells/mL represents the high BTSCC herds.

Proportions and their standard error (SE) were computed for categorical and ordinal variables. Mean and SE were computed for continuous variables. Confidence intervals for proportions were calculated using the normal distribution approximation method (Daniel, 2005). Descriptive statistics were performed using Stata 15 (Stata Corp, College Station, TX, USA). Incidence rate for disease treatment was calculated as: number of new cases divided by total cow time at risk, expressed per 100 milking cow months.

#### Multiple factor analysis and hierarchical clustering

Multiple Factor Analysis (MFA), is a dimensionality reduction approach used for the analysis of several groups of quantitative and/or categorical variables (Bécue-Bertaut & Pagès, 2008). The MFA was performed to summarize the correlation structure of AMD use in adult cows and identify important principal components. The MFA was performed based on a subset of 73 variables (based on the 43 questions) classified into groups of 18 qualitative factors (37 nominal, 15 ordinal, 18 binary variables) and one quantitative factor (three continuous variables—herd size, RHA, and BTSCC). The first two principal components with correlation coefficients (coordinates) of 0.4 or greater were retained for interpretation (Fleiss, Levin & Paik, 2003; Love et al., 2016). The percentage of variance contributed by the group factors to the principal components and the correlation coefficients for the component variables were interpreted. Hierarchical clustering on principal components (HCPC) was performed on the MFA principal coordinates by using the Ward’s criterion to aggregate individual dairies into relatively homogeneous subgroups (clusters) (Bécue-Bertaut & Pagès, 2008; Husson, Josse & Pages, 2010). Separate hierarchical clustering analyses were performed for conventional and organic dairies. The MFA and hierarchical clustering were performed in R statistical software using the “FactoMineR” package (Lê, Josse & Husson, 2008). The identified clusters were then described based on levels of variables that contributed the most percentage of variance. In addition, the demographics and management practices were compared among clusters, specifically, z-test was used for categorical outcomes and Kruskal Wallis test was used for continuous outcomes.

## Results

### Descriptive analyses

**Figure 2:**
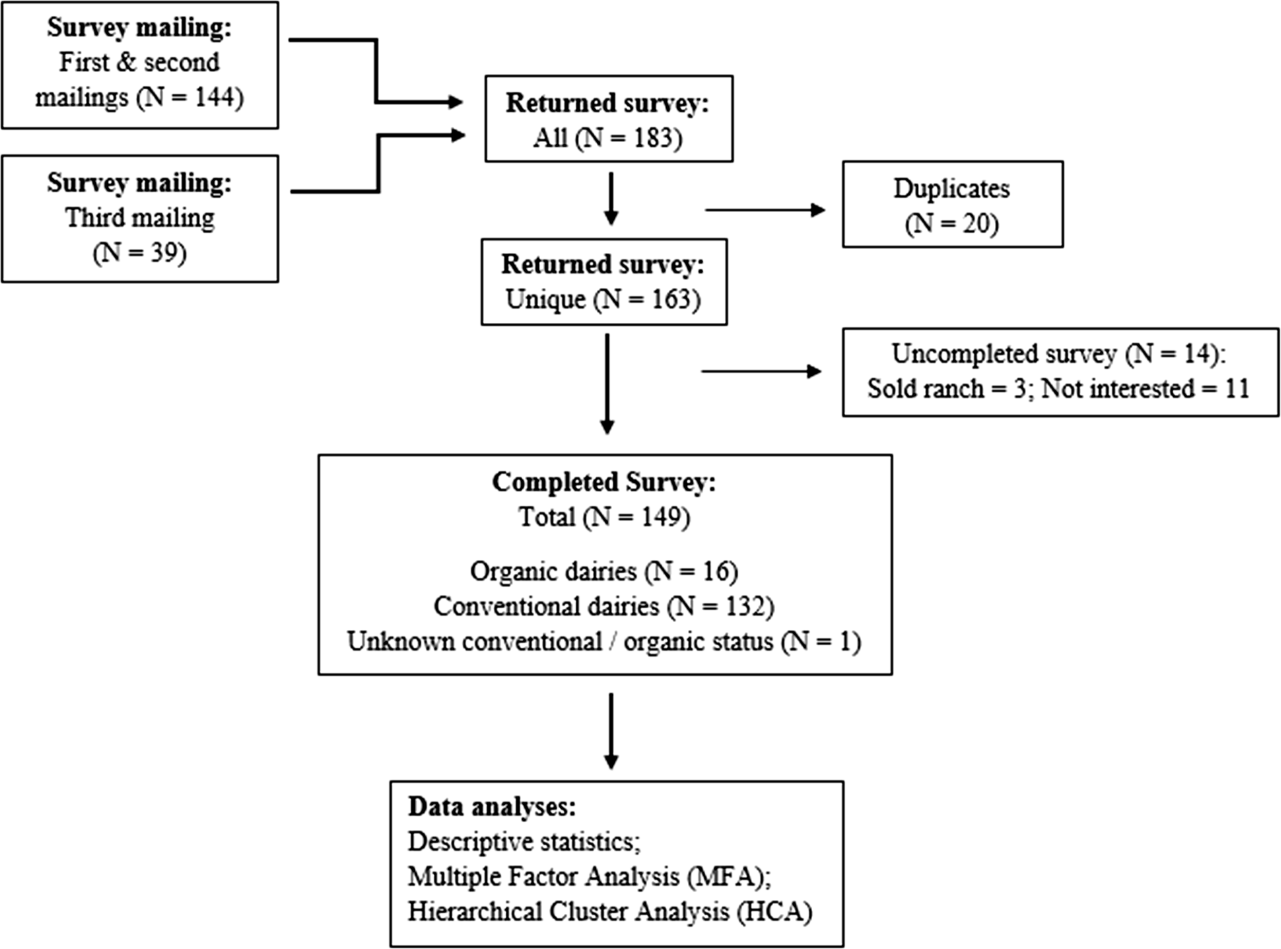
Summary of returned and completed questionnaire on antimicrobial drug use in adult cows mailed to 1,282 licensed grade A California dairy.

A total of 149 (11.6%) survey responses from 19 of the 31 dairy producing counties in CA were received through the three mailings (Fig. 2). Following the distribution of dairy herds statewide (California Department of Food and Agriculture (CDFA), 2018), the greatest proportion of responses came from GSCA, followed by NSJV and NCA. Respondents’ herd characteristics are summarized in Table 1. The overall survey response rate among conventional and certified organic dairies was 11.2% (132/1176) and 15.1% (16/106), respectively. Owners of the dairies constituted the majority of respondents (79%). Additional results are summarized below by management practice, specifically for dry-off practices, vaccination, treatment, drug tracking, drug choices by disease, VCPR, and producers’ perspectives on antimicrobial stewardship.

**Table 1:** Summary of herd information from 149 responses to a mailed questionnaire on antimicrobial drug use in adult cows on California dairies.

Question	*n*	Estimate (%)	SE	95% Confidence limits	
				Lower	Upper
**Respondent role**					
Manager	28	18.9	3.2	13.3	26.1
Owner	95	64.1	3.9	56.0	71.5
Owner-Manger	22	14.8	2.9	9.9	21.6
Veterinarian	3	2.0	1.1	0.6	6.1
**Region**					
Northern California (NCA)	21	14.1	2.8	9.3	20.7
Northern San Joaquin Valley (NSJV)	58	38.9	3.9	31.3	47.0
Greater southern California (GSCA)	70	46.9	4.0	39.0	55.0
**Management**					
Certified organic	16	10.8	2.5	6.3	16.9
Conventional	132	89.2	2.5	83.0	93.6
**Breed**					
Holstein	98	66.2	3.8	58.1	73.4
Jersey	7	4.7	1.7	2.2	9.6
Mixed/Other	43	29.1	3.7	22.2	36.9
**Herd size (# of cow)**					
<1305	82	55.0	4.1	46.9	62.8
1305–3500	58	38.9	3.9	31.3	47.0
>3,500	9	6.0	1.9	3.1	11.2
**Rolling herd average (kg/cow)**					
<10,660	56	41.5	4.2	33.1	50.2
≥10,660	79	58.5	4.2	49.7	66.9
**Bulk tank somatic cell count (cells/mL)**					
<100,000	17	11.5	2.6	7.2	17.7
100,000–199,999	88	59.5	4.0	51.3	67.1
≥200,000	43	29.0	3.7	22.2	36.9

#### Dry cow treatment practices (dry-off protocols)

Overall, 87.2% of the respondents blanket treated cows with intramammary (IMM) AMD and/or teat sealant at dry-off (Table 2). Approximately 42.9% ± 10.7%, 93.1% ± 3.3%, and 95.7% ± 2.4% of the dairies blanket treated dry cows with IMM AMD and/or teat sealant in NCA, NSJV, and GSCA, respectively. However, excluding organic dairies, 75.0% ± 15.3%, 92.9% ± 3.3%, and 95.6% ± 2.4% of the dairies in the three regions reported blanket dry cow treatment. Of all respondents who reported blanket treatment of cows at dry-off, one half (50.0%; 63 conventional dairies) treated with IMM AMD, 43.7% (55 conventional dairies) treated with both IMM AMD and teat sealant, and 6.3% (one organic, seven conventional dairies) treated with teat sealant only. Of all survey respondents, 14.7% reported applying selective dry cow treatment (SDCT) at dry-off. Of these dairies that implement SDCT, one half (50.0%) used both IMM AMD and teat sealant, 45.0% used only IMM AMD, and 5.0% used only teat sealant, when cows are deemed in need of dry cow treatment. However, approximately three quarters (77.2%) of the producers who reported applying SDCT also reported blanket treatment of cows at dry-off, indicating use of both, although the survey instructed to select either blanket treatment or SDCT. A breakdown of classes of AMD used in dry cow treatment on the respondent’s dairies is summarized in Table 2. Regardless of blanket dry cow treatment or SDCT, one half of the respondents used cephalosporins for dry cow treatment, while others used penicillins with or without an aminoglycoside.

**Table 2:** Summary of dry cow treatment practices from 149 responses to a questionnaire on antimicrobial drug use in adult cows on California dairies.

Question	*n*	Estimate (%)	SE	95% Confidence limits	
				Lower	Upper
**Blanket treatment of all dry cows^a^**					
Yes	130	87.2	2.7	80.8	91.7
No	19	12.7	2.7	8.2	19.1
**Blanket treat all dry cows with:**					
Intramammary antimicrobial	63	49.6	4.4	40.9	58.3
Intramammary antimicrobial + Teat sealant	56	44.1	4.4	35.6	52.9
Teat sealant only	8	6.3	2.1	3.1	12.1
**Selective dry-cow treatment (SDCT)**					
Yes	22	14.7	2.9	9.8	21.4
No	127	85.2	2.9	78.5	90.1
**Selective dry-cow treatment with:**					
Intramammary antimicrobial	9	45.0	11.1	24.2	67.7
Intramammary antimicrobial + Teat sealant	10	50.0	11.1	28.1	71.8
Teat sealant only	1	5.0	4.8	0.6	31.1
**Antimicrobial used in dry cow treatment**					
Cephalosporins	65	52.0	4.4	43.1	60.6
Penicillins	32	25.6	3.9	18.6	34.0
Cephalosporins or Penicillins	10	8.0	2.4	4.3	14.3
Cephalosporins or Penicillins or Aminoglycosides	2	1.6	1.1	0.3	6.2
Penicillins or Aminoglycosides	16	12.8	2.9	7.9	19.9

**Notes:**

a Blanket dry cow treatment = an approach to treat every quarter of every cow at drying-off with antimicrobial Cephalosporins = ceftiofur hydrochloride (Spectramast^®^), cephapirin benzathine (Tomorrow^®^) Penicillins = cloxacillin benzathine (Orbenin^®^, Boviclox^®^), penicillin G procaine/novobiocin (Albadry^®^) Penicillins-Aminoglycosides combinations = penicillin G procaine/dihydrostreptomycin (Quartermaster^®^).

#### Dairy cow health management and vaccination practices

The survey showed that 57.7% of respondents provided separate fresh pens, other than the hospital pens, for recently calved cows; 93.9% of the responding dairies’ management included harvesting colostrum from fresh cows to feed newborn calves (Appendix 1). Approximately 85.6% of surveyed dairies vaccinated lactating cows to prevent mastitis due to coliforms, and 7.7% vaccinated to prevent staphylococcus mastitis. Furthermore, 87.0% vaccinated lactating cows to prevent respiratory disease, 73.0% vaccinated lactating cows to prevent abortion and infertility, while one third (35.4%) of dairies vaccinated lactating cows to prevent diarrhea in calves. Additionally, 55.6%, 18.1%, and 0.7% of respondents vaccinated their lactating cows to protect against *Clostridium*, pinkeye, and footrot, respectively.

#### Dairy cow health protocols and antimicrobial drugs treatment practices

The majority of respondents (92.3%) indicated they relied on a veterinarian or a veterinarian in addition to other sources for information about AMD used to treat their cows (Table 3). Other sources for information about AMD included previous experience with the drug (59.0% ± 4.0%), product drug label (40.9% ± 4.0%), drug company material or sales representative (37.5% ± 3.9%), local/national meetings (4.0% ± 1.6%), magazines/industry trade journals (4.0% ± 1.6%), state/county/university cooperative extension (2.6% ± 1.3%), websites (2.6% ± 1.3%), and/or the Food Animal Residue Avoidance Database (1.3% ± 0.9%), (Note: percentages do not add up to 100% due to multiple selections). At the time of our survey, approximately one half of the surveyed dairies, included a veterinarian in their decision on which oral (48.9%) and injectable (51.1%) AMD are purchased and stocked. For producers who did not include a veterinarian in the decision on which oral AMD are purchased or stocked, analysis showed that the majority (92.6% ± 3.1%) had a veterinarian-client-patient relationship (VCPR) for their dairies, while the others relied on a veterinarian for information about AMD used to treat their cows (4.4% ± 2.4%), for cow health monitoring (1.4% ± 1.4%), while one (1.4% ± 1.4%) was an organic dairy. For producers who did not include a veterinarian in the decision on which injectable AMD are purchased or stocked, analysis showed that the majority (91.3% ± 3.6%) had a VCPR for their dairies, while the others relied on a veterinarian for monitoring their cows’ health (6.9% ± 3.3%), and as a source of information about AMD used to treat their cows (1.7% ± 1.7%).

Approximately one third (38.5%) of dairies included a veterinarian in their decision on which AMD is used to treat sick cows, otherwise, decisions were made by the herd manager (68.4% ± 3.8%), dairy owner (54.3% ± 4.0%), treatment crew (16.1% ± 3.0%), and/or milker (0.7% ± 0.6%), (Note: percentages do not add up to 100% due to multiple selection). For producers who did not include a veterinarian in the decision on which AMD is used to treat sick cows, analysis showed that the majority (91.7% ± 2.9%) had a VCPR for their dairies, while the others had a veterinarian observe or discuss the health of their cows (7.1% ± 2.7%) and served as a source of information about AMD used to treat their cows (1.1% ± 1.1%).

**Table 3:** Summary of responses to questions about dairy cow health protocols and antimicrobial treatment practices from 149 responses to a questionnaire on antimicrobial drug use in adult cows on California dairies.

Question	*n*	Estimate (%)	SE	95% Confidence limits	
				Lower	Upper
**Sources info on antimicrobial used to treat cows**					
Veterinarian only	26	18.2	3.2	12.6	25.4
Veterinarian + Others^a^	106	74.1	3.6	66.2	80.6
Others^a^	11	7.7	2.2	4.2	13.4
**Who decides oral antimicrobial to purchase?**					
Include Veterinarian	69	48.9	4.2	40.4	57.5
Dairy personnel only	72	51.1	4.2	42.5	59.6
**Who decides injectable antimicrobial to purchase?**					
Include Veterinarian	66	51.6	4.4	42.5	60.4
Dairy personnel only	62	48.4	4.4	39.5	57.4
**Who decides antimicrobial to treat sick cows?**					
Include Veterinarian	55	38.5	4.0	30.4	46.9
Dairy personnel only	88	61.5	4.0	53.0	69.5
**Have written/computerized health protocol**					
Yes	111	75.0	3.5	67.3	81.3
No	37	25.0	3.5	18.6	32.6
**Who developed the protocols?**					
Include Veterinarian	86	79.6	3.8	70.8	86.3
Dairy personnel only	22	20.4	3.8	13.7	29.1
**Aspect of health for which protocols are used**					
Therapeutic^b^ + prophylaxis^c^	83	76.1	4.0	67.1	83.2
Therapeutic^b^ only	26	23.9	4.0	16.7	32.8
**Included in protocols’ disease-specific treatments**					
Milk and meat withdrawal interval	88	85.4	3.4	77.1	91.0
Milk or meat withdrawal interval	12	11.7	3.1	6.6	19.5
No milk or meat withdrawal interval	3	2.9	1.6	0.9	8.7
**Who has access to animal health protocols?**					
Include Veterinarian	85	77.9	3.9	69.1	84.8
Dairy personnel only	24	22.0	3.9	15.1	30.87
**Trained personnel on treatment protocol for sick cows**					
Yes	94	85.4	3.3	77.4	90.9
No	16	14.5	3.3	9.0	22.5
**Who trained personnel on treatment protocol for sick cows**					
Include Veterinarian	49	52.7	5.1	42.4	62.7
Dairy personnel only	44	47.3	5.1	37.2	57.5
**How often are protocols reviewed/revised?**					
Once to twice a year	62	60.7	4.8	50.8	69.8
Every few years	17	16.6	3.6	10.5	25.3
When a new product is added	23	22.5	4.1	15.3	31.7
**Who reviews/revises health protocols?**					
Include Veterinarian	81	75.0	4.1	65.8	82.3
Dairy personnel only	27	25.0	4.1	17.6	34.1

**Notes:**

a Others = product drug label, drug company material or sales rep, local/national meetings, state/county/university cooperative extension, websites, magazines/industry trade journals, food animal residue avoidance databank, and previous experience with the drug.

b Therapeutic = disease specific treatment.

c Prophylaxis = vaccination, hoof trimming.

*n* = Number of respondents. The questions on “*Have written/computerized health protocol*”, and “*Trained personnel on treatment protocol for sick cows*” has one non-response, while “*Aspect of health for which protocols are used*” and “*Who has access to animal health protocols*” have two non-responses. The questions on “*Who developed the protocols?*”, and “*Who reviews/revises health protocols?*” have three non-responses, while “*Sources info on antimicrobial used to treat cows*” and “*Who decides antimicrobial to treat sick cows?*” have six non-responses. The questions on “*Who decides oral antimicrobial to purchase?*”, and “*Included in protocols’ disease-specific treatments*” have 8 non-responses, while “*How often are protocols reviewed/revised?*” has nine non-responses. “*Who trained personnel on treatment protocol for sick cows?*” has 18 while “*Who decides injectable antimicrobial to purchase?*” has 21 non-responses.

Three quarters (75.0% ± 3.5%) of the respondents indicated having written/computerized animal health protocols (Table 3). In addition, of those who had protocols, approximately four fifths (79.6% ± 3.8%) of the dairies relied on a veterinarian in developing these protocols. Approximately three quarters (76.1% ± 4.0%) of the dairies with protocols have schedules for both disease-specific (92.8% ± 2.4%) and prophylactic interventions (vaccination (74.1% ± 4.1%), hoof trimming (15.1% ± 3.3%); Table 3, percentages do not add up to 100% due to multiple selection). The majority of the surveyed dairies’ disease-specific treatment protocols included milk and meat (84.6% ± 3.5%), milk (9.6% ± 2.8%), or meat (1.9% ± 1.3%) withdrawal intervals (Table 3). Other items included in the treatment protocol included drug dose and drug duration (81.7% ± 3.7%), drug duration (10.5% ± 3.0%), and drug dose (3.8% ± 1.8%); percentages do not add up to 100% due to multiple selection). Multiple personnel (dairy owner (85.7% ± 3.3%), herd manager (81.2% ± 3.6%), treatment crew (52.6% ± 4.7%), nutritionist (15.1% ± 3.3%), office staff (9.8% ± 2.8%)) had access to the animal health protocols.

Approximately 85.4% of the respondents trained their treatment crew members or milkers on treatment protocols for sick cows. A veterinarian was involved in training the treatment crew members or milkers on treatment protocols for sick cows for approximately half (52.7%) of the respondents. Health protocols were reviewed or revised once to twice a year for 60.7% of dairies, when a new product was added for 22.5% of dairies, and every few years for 16.6% of study dairies. Three quarters (75.0%) of the study dairies included veterinarians when reviewing their animal health protocols; the remaining quarter of respondents involved the dairy owner (73.0% ± 4.3%), herd manager (43.2% ± 4.8%), and nutritionist (6.7% ± 2.4%) in protocol review.

#### Antimicrobial drug selection, dosing and tracking practices

Approximately half (50.3%) of the surveyed dairies did not keep a drug inventory log for their dairies (Table 4). For the other half who kept a drug inventory log, the drug-related information they recorded included one or more of: the name of drug (84.7% ± 4.2%); cost (20.8% ± 4.7%); quantity on hand (19.4% ± 4.6%); date of purchase (11.1% ± 3.7%); drug expiration date (11.1% ± 3.7%); or drug supplier/source (9.7% ± 3.4%). Approximately half (51.1%) of these dairies reported recording at least one or two details regarding drug-related information (Table 4). Approximately two thirds (67.6%) of the respondents based their estimation of AMD doses for cows on a combination of multiple factors, including: estimated animal weight and use of manufacturer’s labelled dosage (59.8% ± 4.1%); estimated animal weight and use the dosage prescribed by veterinarian (32.2% ± 3.9%); use of a standard dose by category of animal (11.2% ± 2.6%); estimated animal weight and use of a different dosage than the manufacturer’s label (6.3% ± 2.0%); based on the disease the animal has (5.6% ± 1.9%); and based on how sick the animal appears (4.9% ± 1.8%). Approximately, one third (32.2%) of the respondents’ estimated AMD doses for cows were based on combinations of the previous factors in addition to estimating animal weight and use of the dosage prescribed by the veterinarian. Treatment duration for cows treated with AMD was based on: following veterinarian’s instructions on the prescription label for treatment duration (85.0% ± 3.0%); following the manufacturer’s label treatment duration instructions (57.1% ± 4.1%); stopping the use administration earlier if animals seem to have recovered (7.8% ± 2.2%); extending the use if animals still seem to be sick (3.5% ± 1.5%); or based on previous results using the drug on the farm (1.4% ± 1.0%).

**Table 4:** Summary of responses to questions about antimicrobial drug selection and tracking practices from 149 responses to questionnaire on antimicrobial drug use in adult cows on California dairies.

Question	*n*	Estimate (%)	SE	95% Confidence limits	
				Lower	Upper
**Do you keep a drug inventory log for your dairy?**					
Yes	72	49.6	4.1	41.5	57.7
No	73	50.3	4.1	42.2	58.4
**How many details of drug information^a^ do you record?**					
At least two	17	12.4	2.8	7.8	19.1
Only one	53	38.6	4.1	30.8	47.1
None	67	48.9	4.2	40.5	57.3
**How are antimicrobial doses for cows usually estimated?**					
Veterinarian input	46	32.3	3.9	25.1	40.5
No Veterinarian input	96	67.6	3.9	59.4	74.8
**Treatment duration determination for antimicrobial treated cows**					
Drug labels only	19	13.5	2.8	8.7	20.3
Drug labels + Veterinarian input	61	43.5	4.1	35.5	51.9
Veterinarian input	58	41.4	4.1	33.5	49.8
No drug labels/Veterinarian input	2	1.4	1.0	0.3	5.5
**Factors that influence selection of a second antimicrobial drug**					
Pathogen/drug related	46	36.8	4.3	28.7	45.7
Veterinarian input	46	36.8	4.3	28.7	45.7
Pathogen/drug related + Veterinarian input	33	26.4	3.9	19.4	34.9
**Which antimicrobial treatment information do you track/record?**					
Milk and meat withdrawal interval + Others^b^	65	45.7	4.1	37.6	54.0
Milk or meat withdrawal interval + Others^b^	49	34.5	3.9	27.0	42.7
Others^b^ but not milk or meat withdrawal interval	28	19.7	3.3	13.9	27.1
**How do you track antimicrobial treatments given to cows?**					
Computer + Others^c^	94	64.8	3.9	56.6	72.2
No computer	49	33.7	3.9	26.5	41.9
Memory	2	1.3	0.9	0.3	5.4
**How you track antimicrobial withdrawal period for treated cows**					
Computer + Others^c^	86	61.4	4.1	53.0	69.1
No computer	53	37.8	4.0	30.1	46.2
Memory	1	0.7	0.7	0.1	4.9

**Notes:**

a Details of drug-related information recorded by dairies farmers include name of drug, cost of drugs, quantity on hand, date of purchase, drug supplier/source, and drug expiration date.

b Others include date of treatment, dose, and route.

c Others include paper records kept in barn or office, markings on the animal, and white/chalk board or other temporary markings.

*n* = Number of respondents. The questions on “*D. you keep a drug inventory log for your dairy?*”, and “*How do you track antimicrobial treatments given to cows?*” have four non-responses, while “*How are antimicrobial doses for cows usually estimated?*” and “*Which antimicrobial treatment information do you track/record?*” have seven non-responses. The questions on “*Treatment duration determination for antimicrobial treated cows*”, and “*How you track antimicrobial withdrawal period for treated cows?*” have nine non-responses. “*How many details of drug information do you record?*” has 12 while “*Factors that influence selection of a second antimicrobial drug*” has 24 non-responses.

Selection of a second AMD to treat a sick animal if the first was not successful was based on veterinarian input for approximately two thirds (63.2% ± 4.3%) of the respondents (Table 4). The remaining respondents relied on pathogen/drug related factors: bacterial culture and antibiotic sensitivity results from a laboratory (20.8% ± 3.6%); information outlined in the farm’s protocol for that disease or condition (39.2% ± 4.3%); or experience of previous results using the drug on the farm (18.4% ± 3.4%).

A majority, four-fifths, (80.3% ± 3.3%) of the surveyed dairies confirmed tracking/recording milk, meat, or milk and meat withdrawal intervals along with any of: date, dose, and route of AMD treatment (Table 4). The majority of surveyed dairies tracked the date of treatment (98.5% ± 0.9%); meat withdrawal interval was tracked by 75.3% ± 3.6% of surveyed dairies; AMD treatment dose was tracked by 60.5% ± 4.1% of surveyed dairies; milk withdrawal interval was tracked by 50.7% ± 4.1% of surveyed dairies; milk and meat withdrawal intervals were tracked by 45.7% ± 4.1% of surveyed dairies; while 37.3% ± 4.0% tracked route of AMD treatment. Approximately 64.8% ± 3.9% and 61.4% ± 4.1% tracked AMD treatments given to cows and AMD withdrawal period for treated cows, respectively, using computer software only (9.6% ± 2.4%) or in combination with all or any of markings on the animal (31.0% ± 3.8%), paper records kept in barn or office (46.8% ± 4.1%), and white/chalk board or other temporary marking (7.5% ± 2.1%).

#### Basis and antimicrobial drug choices for disease treatment

##### Mastitis treatment

The statewide mastitis treatment incidence estimated using the survey responses was 2.9 ± 0.3 mastitis cases/100 milking cow months (Appendix 2). The reported regional mastitis treatment incidence was 0.9 ± 0.3, 2.8 ± 0.6, and 3.5 ± 0.4 mastitis cases/100 milking cow months in NCA, NSJV and GSCA, respectively. Approximately one third (36.9% ± 4.2%) of the study dairies relied solely on findings of abnormal milk for their mastitis treatment decision. The remaining producers relied on a combination of factors including abnormal milk (53.0% ± 4.3%), laboratory testing (60.7% ± 4.2%), and treatment while culture is pending then modify treatment if needed (37.6% ± 4.2%). Approximately three quarters (76.7% ± 3.7%) of the study dairies used IMM AMD infusion to individually treat dairy cattle mastitis; a quarter (23.3% ± 3.7%) used both IMM AMD infusion and an oral/injectable AMD. The first choice of AMD for IMM treatment of mastitis was of the cephalosporins class (85.3% ± 3.1%), followed by penicillins (7.3% ± 2.3%), lincosamides (5.6% ± 2.0%), and tetracyclines (1.6% ± 1.1%). The first choice for oral or injectable AMD for mastitis treatment was of the penicillins class (27.7% ± 10.5%), followed by sulfonamides (27.7% ± 10.5%), cephalosporins (22.2% ± 9.7%), and tetracyclines (22.2% ± 9.7%).

##### Metritis treatment

The statewide metritis treatment incidence estimated using the survey responses was 1.9 ± 0.2 metritis cases/100 milking cow months (Appendix 3). The reported regional treatment incidence was 0.5 ± 0.3, 1.3 ± 0.3, and 2.8 ± 0.4 metritis cases/100 milking cow months in NCA, NSJV and GSCA, respectively. Approximately, two thirds (61.3%) of study dairies relied on both clinical presentation (retained placenta (84.0% ± 3.3%), vaginal discharge characteristics (62.1% ± 4.4%), and twins or difficult calving (51.2% ± 4.5%)) and examination (rectal temperature (47.8% ± 4.5%), rectal palpation (30.2% ± 4.2%)) as the basis for the treatment decision. Approximately three quarters (75.6% ± 3.8%) of surveyed dairies used only oral/injectable AMD to individually treat dairy cattle for metritis; 7.3% ± 2.3% used only intrauterine AMD, while 17.0% ± 3.3% used both oral/injectable and intrauterine AMD for treatment of metritis. The first choice of AMD for intrauterine treatment of metritis was the tetracyclines class (52.3% ± 10.8%), followed by cephalosporins (28.5% ± 9.8%), and penicillin (19.0% ± 8.5%). The first choice for oral/injectable AMD for metritis treatment was the cephalosporins class (76.0% ± 4.3%), followed by penicillins (19.7% ± 4.0%), tetracyclines (3.1% ± 1.7%), and sulfonamides (1.0% ± 1.0%).

##### Lameness treatment

The statewide lameness treatment incidence estimated using the survey responses was 1.5 ± 0.2 lameness cases/100 milking cow months (Appendix 4). The reported regional treatment incidence was 1.8 ± 1.6, 0.9 ± 0.1, and 2.0 ± 0.5 lameness cases/100 milking cow months in NCA, NSJV and GSCA, respectively. Approximately half (52.3% ± 4.4%) of the study dairies relied on both lameness signs and hoof trimmer exam for treatment decision, 26.5% ± 3.9% relied on lameness signs only, and 21.0% ± 3.6% relied on hoof examination only. Approximately half (57.0% ± 4.6%) of the study dairies used both hoof treatment (antibiotic foot wrap, heel spray or foot bath) and oral/injectable AMD to treat lameness, 28.9% ± 4.2% used hoof treatment only, and 14.0% ± 3.2% used oral/injectable treatment only. The first choice of AMD for hoof treatment was the tetracyclines class (68.0% ± 6.7%), followed by cephalosporins (23.4% ± 6.1%), lincosamides (4.2% ± 2.9%), and sulfonamides (4.2% ± 2.9%). The first choice for oral/injectable AMD for treatment of lameness was the cephalosporins class (54.0% ± 5.7%) followed by sulfonamides (24.3% ± 4.9%), penicillins (20.2% ± 4.6%), and macrolides (1.3% ± 1.3%).

##### Pneumonia treatment

The statewide pneumonia treatment incidence estimated using the survey responses was 0.3 ± 0.1 pneumonia cases/100 milking cow months (Appendix 5). The reported regional treatment incidence was 0.3 ± 0.2, 0.2 ± 0.1, and 0.5 ± 0.1 pneumonia cases/100 milking cow months in NCA, NSJV and GSCA, respectively. The majority (97.3% ± 1.4%) of surveyed dairies relied on respiratory clinical signs (cough, difficult breathing, nasal discharges) as the basis for treatment decision. Most dairies (98.1% ± 1.2%) used oral/injectable AMD to treat pneumonia in dairy cattle. The first choice for oral/injectable AMD for treatment of adult cattle pneumonia was the cephalosporins class (57.8% ± 4.8%), followed by penicillins (20.5% ± 4.0%), and tetracyclines (7.8% ± 2.6%). Fewer than 6.0% reported using each of the following classes: amphenicols (5.8% ± 2.3%), sulfonamides (4.9% ± 2.1%), fluoroquinolones (1.9% ± 1.3%), and macrolides (0.9% ± 0.9%).

##### Postoperative care

The statewide postoperative care treatment incidence estimated using the survey responses was 0.04 ± 0.01 postoperative care cases/100 milking cow months (Appendix 5). The reported regional treatment incidence was 0.14 ± 0.09, 0.04 ± 0.01, and 0.02 ± 0.01 postoperative care cases/100 milking cow months in NCA, NSJV and GSCA, respectively. Approximately half (49.3% ± 5.8%) of study dairies relied on veterinarian instructions as the basis for AMD treatment in postoperative care, a quarter (27.3% ± 5.2%) treated routinely after displaced abomasal (DA) repair or Caesarian-section (C-section) surgery, while another quarter (23.2% ± 4.9%) reported following either veterinarian instructions or routinely treat after a DA or C-section. The majority of surveyed dairies (96.8% ± 2.1%) used oral/injectable AMD as part of postoperative care. The first choice for oral/injectable AMD for postoperative care was the penicillins class (72.4% ± 5.8%) followed by cephalosporins (25.8% ± 5.7%) and tetracyclines (1.7% ± 1.7%).

#### Veterinarian-client-patient relationship and disease diagnosis practices

Table 5 summarizes the VCPR and disease diagnosis practices of respondents. The majority (91.6% ± 2.3%) of surveyed dairies confirmed they had a VCPR, while approximately one tenth (8.4% ± 2.3%) responded “No” to this question (Table 5). The majority (94.6% ± 1.9%) of the dairies with a VCPR worked with a local veterinarian/clinic while the remaining (5.3% ± 1.9%) worked with a consultant veterinarian. Approximately 65.3% ± 4.1% of the dairies had a written agreement signed with their veterinarian, one fifth (21.5% ± 3.6%) had a verbal agreement with their veterinarian, and 13.0% ± 2.9% had not formally discussed a VCPR but considered they had one based on veterinary care their cows received through their veterinarian. Study dairies indicated that their veterinarian observed, monitored, or discussed the health of the cows with them within a week (40.4% ± 4.1%), every two weeks (36.8% ± 4.0%), monthly (9.9% ± 2.5%), and as needed (12.7% ± 2.8%). Approximately half (47.5% ± 4.1%) of surveyed dairies submitted non-routine samples (e.g. milk culture, placenta, cow for necropsy) to a diagnostic laboratory for diagnosis of infectious diseases in 2018. The majority (73.4% ± 3.6%) of the dairies had used other on-farm diagnostic techniques or procedures, such as culture, auscultation, or lung ultrasound, to guide treatment decisions with AMD for cows.

**Table 5:** Summary of responses to questions about veterinarian-client-patient relationship (VCPR) and disease diagnosis practices , from 149 responses to questionnaire on antimicrobial drug use in adult cows on California dairies.

Question	*n*	Estimate (%)	SE	95% Confidence limits	
				Lower	Upper
**Do you have a veterinarian-client-patient relationship?**					
Yes	131	91.6	2.3	85.7	95.1
No	12	8.4	2.3	4.8	14.2
**Choice of Veterinarian for VCPR**					
Local veterinarian/Clinic	123	94.6	1.9	89.0	97.5
Consultant veterinarian	7	5.3	1.9	2.5	10.9
**Best description of VCPR**					
Written agreement signed by veterinarian and owner	85	65.3	4.1	56.7	73.1
Verbal agreement between veterinarian and owner	28	21.5	3.6	15.2	29.5
Not formally discussed but cows receive veterinary care	17	13.0	2.9	8.2	20.1
**How often vet observe or discuss the health of your cows?**					
Within a week	57	40.4	4.1	32.5	48.7
Forth nightly	52	36.8	4.0	29.2	45.2
Monthly	14	9.9	2.5	5.9	16.1
As needed	18	12.7	2.8	8.1	19.4
**Submitted non-routine samples for infectious disease diagnosis in 2018**					
Yes	69	47.5	4.1	39.5	55.7
No	71	48.9	4.1	40.8	57.1
I don’t know	5	3.4	1.5	1.4	8.0
**Used on-farm diagnostic technique to guide antimicrobial treatment decisions**					
Yes	108	73.4	3.6	65.6	80.0
No	36	24.4	3.5	18.1	32.1
I don’t know	3	2.0	1.1	0.6	6.1

**Notes:**

*n* = Number of respondents. The questions on “*Choice of Veterinarian for VCPR*”, “*Used on-farm diagnostic technique to guide antimicrobial treatment decisions*”, “*Best description of VCPR*”, and “*Submitted non-routine samples for infectious disease diagnosis in 2018*” have one, two, three and four non-responses, respectively. “*D. you have a veterinarian-client-patient relationship?*” has 6 while “*How often vet observe or discuss the health of your cows*” has eight non-responses.

#### Dairy farmer practices and perspectives

Approximately four-fifths (81.8% ± 3.1%) of surveyed dairies participated in animal welfare audit programs (Table 6). The animal welfare audit programs most dairies (97.5% ± 1.4%) participated in were national in scope and included the National Dairy FARM Program (Farmers Assuring Responsible Management), Validus Dairy Animal Welfare Review Certification, and Certified Humane^®^ Program. Personnel on half (52.9% ± 4.2%) of surveyed dairies participated in a dairy quality assurance program in the previous year. Approximately 68.1% ± 3.9% of the survey dairies were familiar with the FDA “MIADs” term, i.e. they recognized that MIADs are further classified as important, highly important or critically important drugs, and are available for livestock only via prescription or VFD pursuant to a VCPR with a licensed veterinarian. The majority (98.6% ± 9.8%) of surveyed dairies were aware that since January 1, 2018, all uses of MIADs in livestock, including injectable AMD such as Penicillin Injectable, Liquamycin^®^ LA 200 (oxytetracycline), and Tylan^®^ Injection (tylosin), and boluses, such as Supra Sulfa^®^ III or Sustain III (sulfamethazine), required a prescription and were no longer sold OTC in CA.

**Table 6:** Summary of practices and perspectives from 149 responses to questionnaire on antimicrobial drug use in adult cows on California dairies.

Question	*n*	Estimate (%)	SE	95% Confidence limits	
				Lower	Upper
**Participate in animal welfare audit programs for dairy farms**					
Yes	122	81.8	3.1	74.7	87.3
No	27	18.1	3.1	12.6	25.2
**Type of animal welfare audit programs**					
National program^a^	119	97.5	1.4	92.5	99.2
Local program^b^	3	2.5	1.4	0.7	7.4
**Participate in dairy quality assurance programs**					
Yes	72	52.9	4.2	44.4	61.2
No	64	47.0	4.2	38.7	55.5
**Type of dairy quality assurance programs**					
National program^c^	13	31.5	7.5	18.5	48.3
Local program^d^	26	68.4	7.5	51.6	81.4
**Familiarity with FDA^e^ MIAD^f^**					
Not sure/Not familiar	44	31.9	3.9	24.5	40.1
Know MIAD available only via prescription	94	68.1	3.9	59.8	75.4
**Aware MIAD require prescription, no more sold OTC^g^ since 2018**					
Yes	141	98.6	9.8	94.5	99.6
No	2	1.4	9.8	0.3	5.4
**Used of OTC and prescription AMD on dairy before 1.1.2018**					
Both OTC and prescription AMD were used to treat cows	93	65.9	3.9	57.6	73.3
Cows were only treated with prescription AMD	22	15.6	3.0	10.4	22.6
Cows were only treated with OTC AMD	5	3.5	1.5	1.4	8.2
Cows were not treated with OTC AMD	15	10.6	2.5	6.4	16.9
Cows were not treated with prescription AMD	2	1.4	0.9	0.3	5.5
Cows were neither treated with OTC nor prescription AMD	4	2.8	1.3	1.0	7.3
**Used of OTC and prescription AMD on dairy before 1.1.2018 (dichotomous response)**					
Cows were treated with OTC AMD ^h^	98	72.6	3.8	64.3	79.5
Cows were not treated with OTC AMD ^i^	37	27.4	3.8	20.4	35.6
**Changes made regarding OTC since 1.1.2018 compared to 2017**					
No changes made	79	56.0	4.1	47.6	64.1
Some changes made	62	43.9	4.1	35.9	52.3
**Use or increased use of alternatives to antimicrobial since 1.1.2018**					
Yes	38	26.5	3.6	19.9	34.4
No	105	73.4	3.6	65.5	80.0
**Made changes to prevent disease outbreak/ spread since 1.1.2018**					
Yes	58	40.8	4.1	33.0	49.1
No	84	59.1	4.1	50.8	66.9
**Antimicrobial drug cost since 1.1.2018 compared to 2017 and earlier**					
Increased	35	24.4	3.5	18.0	32.2
Deceased	41	28.6	3.7	21.8	36.6
No change	67	46.8	4.1	38.7	55.1
**Farm animal health since 1.1.2018 compared to 2017 and earlier**					
Better	43	31.6	3.9	24.3	39.9
Worse	10	7.3	2.2	3.9	13.1
No change	83	61.0	4.1	52.5	68.9

**Notes:**

a National program = National Dairy FARM Program, Validus Dairy Animal Welfare Review Certification, Certified Humane^®^ Program.

b Local program = Dairy Farmers of America.

c National program = National Dairy FARM Program (Validus, Dairy Animal Welfare Review Certification, California Dairy Quality Assurance Program).

d Local program = Creamery, On-farm training, Cooperate extension.

e FDA = U.S. Food and Drug Administration.

f MIAD = Medically Important Antimicrobial Drugs.

g OTC = Over-the-counter.

h Cows were treated with OTC AMD group comprised dairies that used both OTC and prescription AMD to treat cows and dairies that treated cows with only OTC AMD.

i Cows were not treated with OTC AMD group comprised dairies that did not treat cows with OTC AMD and dairies that treated cows with only prescription AMD.

*n* = Number of respondents. The questions on “*Aware MIAD require prescription, no more sold OTC since 2018*”, “*Use or increased use of alternatives to antimicrobial since 1.1.2018*”, and “*Antimicrobial drug cost since 1.1.2018 compared to 2017 and earlier*” have 6 non-responses. “*Made changes to prevent disease outbreak/ spread since 1.1.2018*” has seven non-responses, while “*Used of OTC and prescription AMD on dairy before 1.1.2018*” and “*Changes made regarding OTC since 1.1.2018 compared to 2017*” each has eight non-responses. The question on “ *Familiarity with FDA MIAD*” has 11 non-responses, while “*Participate in dairy quality assurance programs”* and “*Farm animal health since 1.1.2018 compared to 2017 and earlier*” each has 13 non-responses. “*Used of OTC and prescription AMD on dairy before 1.1.2018 (dichotomous response)*” has 14 while “*Type of dairy quality assurance programs*” has 33 non-responses.

The majority (85.2% ± 2.9%) of survey respondents confirmed the use of OTC and/or prescription AMD on their dairies prior to January 1, 2018. Approximately half (56.0% ± 4.1%) of the studied dairies had not made any changes since January 1, 2018 compared to 2017 with regard to injectable and/or IMM AMD that were previously available OTC. Approximately 43.9% ± 4.1% made changes which included: treating fewer animals with AMD (69.3% ± 5.8%); discontinued one or more AMD (43.5% ± 6.2%); use same AMD but the dosage and duration decreased (19.3% ± 5.0%) or increased (3.2% ± 2.2%); added one or more AMD (1.6% ± 1.5%). No respondents indicated treating more animals with AMD after January 1, 2018 compared to 2017. A quarter (26.5% ± 3.6%) of the study dairies confirmed usage or increased use of alternatives to AMD (vitamins (66.6% ± 8.2%), minerals (60.6% ± 8.5%), herbal remedies (36.3% ± 8.3%), and vaccines (36.3% ± 8.3%)) since January 2018. Approximately 40.8% ± 4.1% confirmed making changes in management to prevent disease outbreaks or spread since January 2018; specifically, these changes included: improvements in vaccination programs to prevent disease (83.0% ± 5.1%); quarantine purchased/returning animals from offsite locations (e.g. fairs, shows, calf ranch) (20.7% ± 5.5%); improved biosecurity (e.g. restricted traffic on operation; better isolation of sick animals, or designated separate equipment for feed and manure handling) (11.3% ± 4.3%); and pre-purchase testing of animals before adding to the herd (5.6% ± 3.1%). More than a quarter (28.6% ± 3.7%) of surveyed dairies confirmed decreased AMD costs on their dairies since January 2018 compared to 2017 and earlier. Additionally, 24.4% ± 3.5% and 46.8% ± 4.1% of the dairies confirmed an increase, and no change, respectively, in costs of AMD on their dairies since January 2018 compared to 2017 and earlier. Furthermore, a third (31.6% ± 3.9%) of the surveyed dairies confirmed better animal health on their dairies since January 2018 compared to 2017 and earlier. Only 7.3% ± 2.2% confirmed worse animal health, while 61.0% ± 4.1% confirmed no change in animal health since January 2018 compared to 2017 and earlier.

### Stratified analyses

The percentage of respondents that blanket treated cows with IMM AMD and/or teat sealant at dry-off were comparable across herd sizes; however, respondents whose average number of milking cows were 3,500 or more exclusively blanket treated cows at dry-off (Appendix 6). Conversely, the percentages varied by the region where the dairy is located, approximately half of respondents’ blanket treated in NCA, while approximately 93% and 96% blanket treated in NSJV and GSCA, respectively. Approximately 50%, 81%, and 100% of respondents’ with herd sizes <1,305, 1,305–3,500, and >3,500, respectively, tracked AMD treatments given to cows using computer software only or in combination with all or any of markings on the animal, paper records kept in barn or office, and white/chalk board or other temporary marking. Similar patterns were observed across region, approximately 30%, 54%, and 83% in NCA, NSJV, and GSCA, respectively (Appendix 6). The incidence rates of adult cows treatments per month for mastitis, metritis, or lameness increased as the average number of milking cows in the herd increased. On average, 2.9 ± 0.6, 2.9 ± 0.3, and 3.3 ± 1.4 mastitis cases per 100 adult cows were treated monthly in dairies with herd sizes <1,305, 1,305–3,500, and >3,500 adult cows, respectively (Appendix 6). Across region, 0.9 ± 0.3, 2.9 ± 0.6, and 3.5 ± 0.4 mastitis cases per 100 adult cows were treated monthly in dairies situated in NCA, NSJV, and GSCA, respectively. A greater percentage of producers who included a veterinarian in the decision of which oral AMD to purchase had written or computerized animal health protocols for their dairy compared to producers who did not include a veterinarian in the decision, 87% versus 66% (Appendix 6).

### Multiple factor analysis and hierarchical clustering

The first two principal component dimensions of the MFA explained approximately 14.02% of variability in the survey responses, 9.59% for the first dimension and 4.43% for the second. Seven components with correlation coefficient ≥ 0.4 were identified and retained (Table 7). On the first dimension, four components accounted for 62.7% of the total variance in the data. These components were disease management practices (mastitis, metritis, lameness, and pneumonia management practices), herd demography, AMD usage information, and cow dry-off protocols. On the second dimension, three components (AMD use stewardship practices, familiarity with FDA “medically important antimicrobial” term, and producer perceptions of AMD on dairies) accounted for 43.1% of the total variance in the data. The cluster analysis of conventional dairy participants partitioned study dairies into two possible clusters (cluster C1 and cluster C2) based on the height of the dendrogram; while the analysis of organic dairy participant grouped into a single cluster.

**Table 7:** Summary of multiple factor analysis showing seven identified components extracted from 18 antimicrobial drug use variables collected from 149 responses to a questionnaire distributed to grade A licensed California dairies.

Identified components	Variation proportion (%)	Component variables	Correlation
**Disease management practices**	35.1		
*a. Mastitis management practices*	*10.2*	Mastitis: Basis for treatment decision	0.576
		Mastitis: Treat with intramammary AMD infusion	0.695
		Mastitis: Treat with intramammary and oral/injectable AMD	0.694
*b. Metritis management practices*	*9.0*	Metritis: Basis for treatment decision	0.522
		Metritis: Treat with intrauterine/oral/injectable AMD	0.609
*c. Lameness management practices*	*8.7*	Lameness: Basis for treatment decision	0.483
		Lameness: Hoof treatment (wrap/spray/bath), or oral/injectable	0.504
*d. Pneumonia management practices*	*7.2*	Pneumonia: Treatment bolus/injectable treatment	0.505
AMD use stewardship practices	15.6	Administration of appropriate AMD, dose, route and duration	0.430
		Good record keeping on treatments and treatment dates	0.456
		Having a current veterinarian-client-patient-relationship (VCPR)	0.415
		Observing withdrawal periods and drug residue avoidance	0.491
		Using alternatives to AMD (vaccines, minerals, vitamins, herbal remedies)	0.426
Familiar with FDA^a^ “MIADs^b^” terms	14.1	Use of OTC^c^ and prescription AMD on your dairy before 01.2018	0.502
Herd demography	13.5	Conventional vs. certified organic dairies	0.609
		Annual rolling herd average for milk production	0.612
Producer perceptions of AMD on dairies	13.4	AMD use in livestock does not cause problems in humans	0.415
		Any use of AMD may result in infections that are more difficult to treat in the future	0.400
AMD usage information	7.8	How do you track AMD treatments given to cows?	0.476
		How do you track AMD withdrawal for treated cows?	0.557
Dry-off protocols	6.3	Classes of AMD used at dry-off	0.446

**Notes:**

a FDA = U.S. Food and Drug Administration.

b MIAD = Medically Important Antimicrobial Drugs.

c OTC = over-the-counter.

### Conventional dairy clusters

The profiles of the conventional dairy clusters are described in Table 8, while their comparison to the organic dairy cluster is described in Appendix 7. Herd size varied between the clusters with the largest herds in cluster C1. Cluster C1 included dairies from the three regions and all breed types; while C2 included dairies in NSJV and GSCA region, mainly Holstein and herds with more than one breed.

**Table 8:** Description of the two typologies identified using Multiple factor analysis and Hierarchical clustering and allocation of conventional surveyed dairies (values with different superscript letters in a row are significantly different (*p* < 0.05)).

Components	Characteristics	Cluster C1 % ± SE	Cluster C2 % ± SE
		*N* = 122	*N* = 10
Herd demography	**Region**		
	Northern California	6.6 ± 2.2^a^	0.0 ± 0.0^a^
	Northern San Joaquin Valley	43.4 ± 4.4^a^	40.0 ± 15.4^a^
	Greater Southern California	50.0 ± 4.5^a^	60.0 ± 15.4^a^
	**Breed**		
	Other	26.4 ± 4.0^a^	30.0 ± 14.4^a^
	Hostein	69.4 ± 4.1^a^	70.0 ± 14.4^a^
	Jersey	4.1 ± 1.8^a^	0.0 ± 0.0^a^
	**Population**		
	Herd size (median)	1,265.0^a^	715.0^a^
	Rolling herd average (median)	11,339.8^a^	10,092.4^b^
Dry-off protocol	**Blanket^1^ treatment of all dry cow**		
	No	4.9 ± 1.9^a^	20.0 ± 12.6^b^
	Yes	95.1 ± 1.9^a^	80.0 ± 12.6^b^
	**AMD^2^ used in dry cow treatment**		
	Cephalosporins	50.9 ± 4.6^a^	66.7 ± 15.7^a^
	Penicillins	27.1 ± 4.1^a^	0.0 ± 0.0^b^
	Cephalosporins or Penicillins	10.5 ± 2.8^a^	0.0 ± 0.0^a^
	Penicillins or Aminoglycosides	11.4 ± 2.9^a^	33.3 ± 15.7^b^
Disease management	**Mastitis: Basis for treatment decision**		
	Findings of abnormal milk	34.5 ± 4.3^a^	100.0 ± 0.0^b^
	Abnormal milk + Lab testing	25.2 ± 3.9^a^	0.0 ± 00^a^
	Abnormal milk + Lab testing + Treat pending test result	40.3 ± 4.4^a^	0.0 ± 0.0^b^
	**Mastitis: Treat with AMD**		
	No	1.6 ± 1.1^a^	25.0 ± 15.3^b^
	Yes	98.4 ± 1.1^a^	75.0 ± 15.3^b^
	**Metritis: Choice of AMD treatment**		
	Bolus/Injectables	75.2 ± 3.9^a^	100.0 ± 0.0^a^
	Intrauterine	6.8 ± 2.3^a^	0.0 ± 0.0^a^
	Intrauterine + Bolus/Injectables	17.9 ± 3.5^a^	0.0 ± 0.0^a^
	**Lameness: Choice of AMD treatment**		
	Hoof treatment (antibiotic wrap, heel spray, foot bath)	28.4 ± 4.3^a^	66.7 ± 27.2^a^
	Bolus/Injectables	14.7 ± 3.3^a^	0.0 ± 0.0^a^
	Hoof treatment + Bolus/Injectables	56.8 ± 4.7^a^	33.3 ± 27.2^a^
Antimicrobial stewardship	**How do you track AMD treatments given to cows?**		
	Computer + Others^3^	69.7 ± 4.1^a^	40.0 ± 15.4^b^
	No computer	30.3 ± 4.1^a^	60.0 ± 15.4^b^
	**How you track AMD withdrawal period for treated cows**		
	Computer + Others^3^	66.4 ± 4.2^a^	22.2 ± 13.8^b^
	No computer	33.6 ± 4.2^a^	77.8 ± 13.8^b^
	**Administration of appropriate AMD, dose, route, duration**		
	Very important	97.5 ± 1.4^a^	80.0 ± 17.8^b^
	Some importance	2.5 ± 1.4^a^	20.0 ± 17.8^b^
	Not important	0.0 ± 0.0^a^	0.0 ± 0.0^a^
Antimicrobial use on dairies	**Used of OTC^4^ and prescription AMD on dairy before 1.1.2018 (dichotomous response)**		
	Cows were treated with OTC AMD	72.3 ± 4.1^a^	66.7 ± 27.2^a^
	Cows were not treated with OTC AMD	27.7 ± 4.1^a^	33.3 ± 27.2^a^
Producer perceptions of AMD on dairies	**Any use of AMD may result in infections more difficult to treat in future**		
	Strongly agree/agree	11.6 ± 2.9^a^	40.0 ± 21.9^b^
	Neutral	30.6 ± 4.2^a^	20.0 ± 17.8^a^
	Strongly disagree/disagree	57.9 ± 4.5^a^	40.0 ± 21.9^a^

**Notes:**

1 Blanket dry cow treatment = an approach to treat every quarter of every cow at drying-off with antimicrobial.

2 AMD = Antimicrobial drug.

3 Others = markings on the animal, paper records kept in barn or office, white/chalk board or other temporary marking.

4 OTC = over-the-counter.

Most of the cluster C1 dairies (95.1%) implemented blanket treatment of dry cows with IMM AMD and teat sealant, compared to dairies in cluster C2 (80.0%). Notably, dairies in cluster C1 used multiple AMD classes (cephalosporins, penicillins, and aminoglycosides) for dry cow treatment, dairies in cluster C2 used mainly cephalosporins. Approximately two thirds of the dairies in cluster C1 tracked both AMD treatments given to cows (69.7%) and withdrawal periods for AMD treated cows (66.4%) using a computer, in addition to other methods. The reverse was true for dairies in cluster C2, approximately two fifths (60.0%) do not track AMD treatments and two thirds (77.8%) do not track AMD withdrawal periods using a computer.

For mastitis treatment, dairies in cluster C1 relied mostly on clinical signs and laboratory testing, and tended to treat cows while culture results, if any, were pending. Dairies in cluster C2 relied mostly on clinical signs. Most C1 dairies (98.4%) treat mastitis with AMD, compared to 75.0% in C2. For metritis treatment, dairies in cluster C2 totally relied on oral/injectable AMD, while cluster C1 relied on both oral/injectable AMD and intrauterine treatment. Similarly, dairies in cluster C2 managed lameness in adult cattle using mostly hoof treatment and less of oral/injectable AMD compared to dairies in C1.

Most dairies in cluster C1 (97.5%) strongly agreed that administration of appropriate AMD, dose, route and duration are important AMD stewardship practices compared to less in C2 (80.0%). However, responding to questions relating to AMD resistance, more dairies in cluster C2 (40.0%) strongly agreed with the statement “*Any use of antibiotics may result in infections more difficult to treat in the future*”, whereas, fewer dairies in cluster C1 (11.6%) agreed with same statement.

### Organic dairy cluster

All the survey’s organic dairies formed a single cluster characterized by multiple breeds with Holstein and Jersey making up 37.5% and 12.5%, respectively and the remaining majority being other breeds. The dairies in the cluster were predominantly in NCA (81.2%) with three respondent in GSCA (18.8%), had a median herd size of 325 milking cows, and a median annual rolling herd average of 8,235 kg per cow. Two organic dairy (12.5%) reported use of blanket treatment of dry-cows depending on the season with an external teat sealant, one organic dairy (6.3%) reported use of blanket treatment of dry-cows with organic intramammary infusion (Phyto-Mast, Bovin-ity Health LLC, Narvon, PA). One organic dairy (6.3%) reported use of selective dry-cow treatment with an organic intramammary infusion (Phyto-Mast), and one other dairy reported use of selective dry-cow treatment with external teat sealant depending on season and mastitis history. Approximately two thirds (60.0%) of the organic dairies reported use of abnormal milk and sample testing at a milk quality laboratory as basis for mastitis treatment decisions, the remaining relied on abnormal milk findings only. Of the organic dairy respondents, 18.8% indicated treatment of mastitis with AMD, while 50% reported treatment of metritis with intrauterine bolus, and 100% reported treatment of lameness with bolus or injection of AMD and hoof treatment. Few organic dairies in the cluster used computers to track AMD treatments (41.7%) and withdrawal periods (37.5%). Three fifths (60.0%) of the survey’s organic dairies indicated use of OTC AMD, or OTC and prescription AMD prior to January 1st 2018. The remaining organic dairy responses (40.0%) indicated no OTC AMD, prescription but no OTC AMD, neither OTC nor prescription, or no response.

Most of the organic respondents (76.9%) identified the following AMD stewardship practices as very important: administration of the appropriate dose, route and duration of AMD; good record keeping of treatments and treatment dates; and observing withdrawal periods and drug residue avoidance. However, on a statement that any use of AMD may result in infections more difficult to treat in future, 38.5% of the survey’s organic dairies agreed or strongly agreed, 38.5% disagreed or strongly disagreed, while 23.0% were neutral.

## Discussion

The purpose of the current study was to explore the antimicrobial stewardship practices, AMD use and health management practices in adult cows on CA dairies following the passage of California SB 27 and FDA’s VFD rules on the judicious use of MIADs in food producing animals. While the current survey achieved an 11.6% response rate, the responses represented 19 (61%) of the dairy producing counties in CA, responsible for over 94.9% of total CA milk production. Producers who participated in our survey were distributed across the three milk sheds in CA, specifically 14% in NCA, 39% in NSJV, and 47% in GSCA. The survey respondent’s distribution was similar to the distribution of dairy operations across CA published by the CDFA, California Dairy Statistics 2017 (California Department of Food and Agriculture (CDFA), 2018) which reported the number of dairies in NCA as 16%, NSJV as 40%, and GSCA as 44%. Similar to Love et al. (2016), our survey identified most of the state’s organic dairies as located in NCA. In CA, previous studies have identified three distinct milk sheds based on diversity of dairy operations shown to have differences in calf management and disease prevalence, such as bovine respiratory disease (Karle et al., 2019; Maier et al., 2019). Our study identified seven major factors driving the diversity in AMD use and treatment practices in adult cows on CA dairies.

Additional survey findings categorized CA dairy producers into three clusters characterized by demographic parameters, dry-off protocol, disease management, and AMD stewardship practices. Our findings identified important management practices, such as dry cow treatment practices, cow health protocols and treatment practices, where extension and outreach efforts should focus on improving antimicrobial stewardship. One of the goals of such extension education is to introduce and reinforce preventive measures that may reduce the use of AMDs on dairies, including vaccines, supplements, and preventive measures, such as internal teat sealants at dry-off and hoof trimming (Ekakoro et al., 2018; Golder, Hodge & Lean, 2016; Higgins et al., 2017; Sharma et al., 2018). Other goals of an extension program are to reduce the risk of antimicrobial resistance when treatments are necessary through use of effective treatment protocols based on veterinary input presented and explained to dairy staff during at least annual training. The appropriate use of AMD in terms of drug class, route, dose and duration, and treatment records and withdrawal tracking are also integral to a dairy’s antimicrobial stewardship practices.

### Dry cow treatment practices (dry-off protocols)

Our findings indicated that the majority of surveyed dairies (93.7%) treated all end-of-lactation cows presenting for dry-off with IMM AMD and/or teat sealants. About half (49.6%) of the respondents used IMM AMD, 44.1% used both IMM AMD and teat sealant, while 6.3% used only teat sealant at dry-off. The USDA-National Animal Health Monitoring System 2014 Dairy Study, (National Animal Health Monitoring System (NAHMS), 2016a), showed that 86.1% of dairy operations in the Western US (California, Colorado, Idaho, Texas, and Washington), and 79.7% in the Eastern US (Indiana, Iowa, Kentucky, Michigan, Minnesota, Missouri, New York, Ohio, Pennsylvania, Vermont, Virginia, and Wisconsin) treated all their cows at dry-off with IMM AMD, while 90.8% of the operations used some antimicrobials on at least some of the cows at dry-off. In contrast, 7.2% and 9.4% of the operations in the Western and Eastern US, respectively, did not use IMM AMD at dry-off. According to our survey, 14.7% of respondent dairies carried out selective dry cow treatment at dry-off; this estimate was close to the 13.9% and 20.3% reported by NAHMS for Western and Eastern US, respectively. The majority of the study dairy operations (61.6%) used the cephalosporins (Spectramast^®^, Tomorrow^®^) class of AMD in dry cow treatment, 33.6% used AMD containing penicillins (Orbenin^®^, Boviclox^®^, Albadry^®^), while 14.4% used penicillin-aminoglycoside combinations (Quartermaster^®^). Dry cow treatment statistics based on the NAHMS 2014 dairy study showed comparable estimate for the use of cephalosporins (63.3%) on Western US dairy operations (29% used Cefa-Dri^®^/Tomorrow (cephapirin: first-generation cephalosporins), while 34.3% used Spectramast^®^ (ceftiofur: third-generation cephalosporins). The contrast was seen with use of AMD containing penicillins on dry-off treatment which was reportedly greater in our study 33.6% compared to the 26.8% (Boviclox^®^, Dry-clox^®^, Orbenin^®^, Albadry^®^ plus) in NAHMS 2014 Dairy study; and with Quartermaster^®^ which was less in our study (14.4% compared to 24.0% in NAHMS 2014 Dairy study) (National Animal Health Monitoring System (NAHMS), 2016a). Several studies have described the benefits of dry cow therapy to udder health (Nickerson & Oliver, 2014; Owens et al., 2001; Vilar et al., 2018). The practice prevents mastitis in the early dry period, allows milk-producing tissue to redevelop in cured quarters, and reduces clinical mastitis at freshening. Blanket dry cow therapy has been used to treat cases of chronic mastitis caused by *Staphylococcus aureus* and *Streptococcus agalactiae* and to reduce new IMM infections early in the dry period (Bonsaglia et al., 2017). However, blanket treatment of all dry cows exposes cows without a history of mastitis to unnecessary antimicrobial use (Bonsaglia et al., 2017; Higgins et al., 2017). Recent studies have demonstrated the benefits of selective dry cow therapy as an effective alternative to blanket treatment with AMD (Bonsaglia et al., 2017; Cameron et al., 2015, 2014; Scherpenzeel et al., 2014). Some countries such as Finland, the Netherlands, and Denmark have banned blanket dry cow treatment (Bennedsgaard, Klaas & Vaarst, 2010; Scherpenzeel et al., 2016; Vilar et al., 2018). Selective dry cow treatment has been shown to result in: decreased use of antimicrobials for IMM infections during the dry period by as much as 21%, resulting in reduced incidence of AMR, and thus translating to a significant reduction in production cost (Cameron et al., 2014; Østerås, Edge & Martin, 1999). However, determination of an effective tool to identify cows that will benefit from selective dry cow treatment could be challenging.

### Dairy cow health management and vaccination practices

Most dairy producers in the survey vaccinated lactating cows to prevent mastitis due to coliform bacteria (85.6%), respiratory disease (87.0%), and abortion and infertility (73.0%). Furthermore, 95.3% of the study dairies administered at least one type of vaccine to lactating cows for disease prevention. This percentage is higher than the 73.8% of dairy operations that administered any vaccine to cows for disease prevention reported by National Animal Health Monitoring System (NAHMS), (2016b), including, 18.1% of U.S. operations that vaccinated lactating cows against coliform mastitis (range, 2.4–50.8% depending on herd size) (National Animal Health Monitoring System (NAHMS), 2016a). Generally, NAHMS reported that more dairy operations in the western U.S. (86.3%) vaccinated their lactating cows for any disease compared to dairies in eastern U.S (72.6%) (National Animal Health Monitoring System (NAHMS), 2016b). Several other studies have highlighted the benefits of vaccination in preventing or reducing the severity of several infections in vaccinated cows (Ferguson, Galligan & Cortese, 1997; González et al., 1989; Gorden et al., 2018; Pereira et al., 2013; Prado et al., 2011) and their offspring (Dubrovsky et al., 2019a, 2019b; Maier et al., 2019). Scientific efforts should be expanded towards the development of alternative non-AMD disease treatment and prevention approaches geared towards reducing the incidence of AMR in livestock production.

### Dairy cow health protocols and antimicrobial treatment practices

Overall, 92.3% of the study dairy producers relied on a veterinarian only or veterinarian and other sources for information regarding AMD to use for treatment of adult cows. Half (49.3%) of the dairies included a veterinarian in their decision on which oral and injectable AMD are purchased and stocked, while 37.9% included a veterinarian in their decision on which AMD is used to treat sick cows. These proportions are consistent with the National Animal Health Monitoring System (NAHMS) (2018) survey which confirmed that 96.2% of U.S. dairy producers consulted a veterinarian or a drug label created by a veterinarian in their treatment decisions, 55.1% consulted with a veterinarian or the drug label created by the veterinarian for their decision on what drug to use while 44.0% relied on previous experience with the drug. From a 2002 survey of antibiotic usage on 113 dairy herds from 13 counties in Pennsylvania (Sawant, Sordillo & Jayarao, 2005), only 32% of dairy producers sought veterinarian advice before administering antibiotics to cows on their dairies, a proportion similar to what was observed in our survey. The above data suggests the need for increased veterinarian involvement with producers in their treatment decisions, in line with state regulation (FAC 14400–14408 (formerly SB-27)) which require that all MIADs, other than those intended to be fed to livestock (which require a VFD), may only be purchased and administered with a prescription from a California licensed veterinarian within a valid VCPR.

In our study, 75.0% of dairies confirmed having written/computerized animal health protocols for cows, mainly developed with veterinarian inputs for both treatment and preventative therapeutic uses. In a 2007 study aimed at determining South Carolina (SC) dairy farmers’ knowledge on the importance of prudent antibiotic use on dairy cows, Friedman et al. (2007) reported that only 32% of participating farms had written protocols for diagnosing and treating common medical conditions in the dairy herd. Sawant, Sordillo & Jayarao (2005) reported only 21% of dairy producers had written plans for treating sick animals. In the SC study, most farmers expressed that though their protocols may have been ‘unwritten’, they followed standard operating procedures in dairy herd management. In a survey of dairy herds in Washington State and Pennsylvania, Raymond, Wohrle & Call (2006) and Sawant, Sordillo & Jayarao (2005) concluded that the lack of written protocols for disease treatment on dairies could lead to inappropriate use of antibiotics which can promote the selection of resistant bacteria. Although, a high percentage (75.0%) of farmers in the current CA study confirmed having written/computerized animal health protocols compared to the Washington and Pennsylvania’s studies, research is needed to investigate this association further.

### Antimicrobial drug selection, dosing and tracking practices

Drug inventory and treatment records help dairy producers track drug use and treated cows. Approximately half of the surveyed dairies reported not keeping a drug inventory log on their dairies, information which otherwise may help the producers monitor drug use on the dairy over time, track availability and stocking of drugs to avoid shortages or waste due to their expiration, and prevent accidental administration of expired drugs. A survey of dairies in Pennsylvania found only 50% of the dairies kept written or computerized records of antibiotic treatment that could be verified (Sawant, Sordillo & Jayarao, 2005). Other important benefits of a drug inventory to producers include the opportunity to monitor drug label changes over time and monitor costs incurred for AMDs over time and by drug class. Similarly, maintaining adequate treatment records benefits food safety, supports improved management of drugs used that could help prevent drug residues in milk and meat, helps ensure an effective herd health plan, and reduces farmer liability or violations during regulatory follow-up.

At least 36.8% of our survey respondents relied on bacterial culture and antibiotic sensitivity results in choosing a second antibiotic drug to treat a sick animal if the first was not successful. In addition to consulting with a veterinarian, laboratory testing may be a better approach after a treatment failure to confirm the bacterial etiology or its antimicrobial sensitivity if the diagnosis was correct.

Most (80.3%) of the dairies in our survey confirmed tracking/recording milk, meat, or milk and meat withdrawal intervals. According to NAHMS, more than 90.0% of U.S. dairy operations administered drugs that required a milk withdrawal period or a milk and meat withdrawal period (National Animal Health Monitoring System (NAHMS), 2016a). The National Milk Producers Federation (NMPF) recommends that producers keep written or electronic records on all cattle treated with drugs that require a withdrawal period for milk or meat, records should be maintained for at least two years or as otherwise required by Federal or State law, and should be accessible to everyone who works with the animals (National Milk Producers Federation (NMPF), 2019). Approximately 35% of dairies in our survey tracked milk or meat withdrawal intervals but not both; such a practice is not uncommon, as certain drugs in specific formulations (such as Ceftiofur) and that are used in adult cow treatment may only have meat and no milk withdrawal periods. Similarly, approximately 20% (28/142) of surveyed dairies did not track milk or meat withdrawal periods; 6 (20%) of these dairies were organic while the remaining 80% were conventional dairies. Not tracking milk or meat withdrawal period in this group could be related to not using AMDs on these dairies or may represent cases of drugs used that do not have a milk withdrawal period.

### Basis and antimicrobial choices for disease treatment

According to the survey, the number one reason for AMD use on CA dairies was for mastitis treatment, followed by metritis, lameness, pneumonia, and postoperative care; similar to the report by Sawant, Sordillo & Jayarao (2005) and Raymond, Wohrle & Call (2006). Approximately, three mastitis cases per 100 milking cow months were reported based on clinical signs and laboratory diagnosis. Based on NAHMS 2014 survey, 99.7% of dairy producers in 2013 reported having at least one case of mastitis, and one-fourth of all cows on their dairies had clinical mastitis (National Animal Health Monitoring System (NAHMS), 2016a). Two thirds of dairies in our survey individually treat mastitis cows with IMM AMD infusion, while the remaining used IMM infusion and systemic AMD, which is similar to the estimate reported in the NAHMS 2014 dairy study where 89.4 % of dairy operations used IMM AMD to treat mastitis. Cephalosporins remained the primary antimicrobials for treating mastitis, followed by penicillins, lincosamides, and tetracyclines as also evident from the NAHMS 2014 survey.

According to NAHMS 2014 dairy study (National Animal Health Monitoring System (NAHMS), 2018), 58.8%, 61.0%, and 54.1% of dairy operations treated some cows with antimicrobials for reproductive disease, lameness, and respiratory disease, respectively. In our survey, approximately 2 per 100 adult cows were treated for each of metritis and lameness per month, and 0.3 per 100 adult cows were treated for pneumonia. Tetracyclines were the primary antimicrobials used for intrauterine treatment of metritis and cephalosporins for systemic treatment. Lameness was managed in survey dairies with hoof treatment and systemic antibiotics, primarily with tetracyclines and cephalosporins.

### Veterinarian-client-patient relationship (VCPR) and disease diagnosis practices

The basic elements of a VCPR are that the veterinarian agreed to assume the responsibility for the animal’s health and the producer agreed to comply with the veterinarian’s instructions; the veterinarian has sufficient knowledge of the animals to make a general diagnosis and assumes responsibility for follow-up care, including adverse reactions and/or treatment failures; and the veterinarian maintains all animal records (16 CCR 2032.1). Most dairies (92%) in our survey confirmed having a valid VCPR for their dairies. Further analysis of the survey questions revealed that the 8.0% (12/143) of producers who said they do not have a VCPR for their dairies may have either misunderstood the question, were not familiar with the term, or mistakenly selected the incorrect answer, as their responses to other questions relating to cow health on their farms showed direct involvement of a veterinarian in cow health care decisions. Specifically, of the 12 producers who indicated not having a VCPR, 10 included veterinarians as one of the sources they relied on for information about AMD used to treat cows. The remaining two dairies had either submitted non-routine samples for infectious disease diagnosis or had used other on-farm diagnostic techniques to guide treatment decisions with AMD for cows; both diagnostic activities were most likely associated with a veterinarian. Hence, continued education of dairy producers is necessary to update their understanding of the requirements of the current law and what a valid VCPR entails.

### Dairy farmer practices and perspectives

Animal welfare audit programs are designed to improve welfare standards in livestock operations by ensuring animals are humanely raised. Dairy quality assurance programs, on the other hand, are designed to improve product quality through assessments and monitoring, thereby promoting consumer health, environmental health, and dairy cattle health and welfare. Most dairies in our survey participated in welfare (97.5%) and quality assurance programs (52.9%) in the previous year, highlighting the dedication of CA producers to maintaining high standards of animal welfare and product quality. Based on the NAHMS 2014 dairy study, approximately 45.9% of all dairy operations nationwide participated in a quality assurance program, with higher participation by operations in Western US (66.8%) compared to in the Eastern US (43.9%) (National Animal Health Monitoring System (NAHMS), 2018). The majority (98.6%) of producers in our survey were aware that all uses of medically important AMD in livestock require a VFD or prescription. Such a high percent shows the successful impact of SB-27 legislation on responding CA dairies and producers, the majority of whom had used OTC AMD on their dairies prior to January 1, 2018 and have made changes, such as, decreasing usage of AMD which were previously available OTC, increased usage of alternatives to AMD, and introduced management changes leading to better herd health as reported by a third of the surveyed dairies.

### Multiple factor analysis and hierarchical clustering

The MFA identified seven components that accounted for 99.7% of the variation in the data. The seven components identified the important questions that could be considered for a risk assessment tool for AMD use and AMR on CA dairies in future studies, similar to the approaches adopted for bovine respiratory disease (Love et al., 2016; Maier et al., 2019) and Johne’s disease (Berghaus et al., 2005) risk assessment.

Cluster C1 consisted of large-scale conventional dairies (median herd size: 1,265 cows; median RHA: 11,340 kg/cow) situated mainly in the GSCA and NSJV region. Dairies in this cluster were characterized by their AMD usage in dry cow management (blanket treatment of all dry cows) at the end of each lactation, and in the management of disease conditions such as mastitis, metritis, and lameness in adult cows. The cluster can be linked with good knowledge of AMD stewardship practices as evident by the high frequency of AMD treatment and withdrawal tracking, most often using a computer, and overall agreement to statements regarding drug use stewardship practices and AMR in their dairy operations.

Cluster C2 consisted of mid-sized conventional dairies (median herd size: 715 cows; median RHA: 10,092 kg/cow) located in NSJV and GSCA regions. Producers in this cluster could be linked with some knowledge of AMD use stewardship practices as characterized by their high level of agreement (100%) to statements regarding AMD use stewardship practices. They tend to show more agreement regarding AMR issues raised in the study compared to dairies in clusters C1. Producers in this cluster could benefit from outreach and education relating to use of computer in record keeping, AMD use stewardship practices and their impact on AMR in dairy operations.

The organic cluster consisted of small-sized dairies mainly located in NCA and reported little or no usage of AMD in their dairy operations. Organic dairies do not use AMD amongst other prohibited chemicals (such as pesticides, hormones with exception of oxytocin) unless sick animals require antibiotics. Emergency AMD treatments to maintain the welfare of the sick animals are allowed on organic dairies; however, such animals have to be segregated from the organic herd and sold to a non-organic market in addition to strict documentation and reporting requirements (Code of Federal Regulations (CFR), 2011). The survey’s organic dairies had mixed responses on AMD stewardship practices with approximately two-fifths agreeing that any use of AMD may result in infections more difficult to treat in the future.

The current study data were collected based on voluntary participation, in accordance with the SB 27 from CA licensed grade A dairy producers. The mean dairy herd size in our survey was 1,550 cows per herd, which was greater than a 2017 estimate of average herd size of 1,304 cows per herd (California Department of Food and Agriculture (CDFA), 2018); which may suggest over representation of large herds in our survey. However, response rates across regions in our survey were similar to the regional distribution of herds published in the California Department of Food and Agriculture (CDFA) (2018) report. Therefore, potential bias due to regional difference was minimal as all 3 regions were proportionately represented in our survey. In addition, survey responses may reflect respondent recall and/or reporting bias and may not be representative of the actual practices on their dairies. Furthermore, AMD usage and treatment practices in respondent dairies represented the period of time shortly after the implementation of SB 27 on January 2018 and so may not represent changes that have taken place since the survey was administered. Hence, a follow-up survey is necessary to capture the long-term impact of possible changes in stewardship practices among CA dairy producers. Additional studies are needed to measure factors associated with herd demography, drug use treatment practices, and changes in management and stewardship practices since SB-27. Further studies correlating AMR profile of dairies with levels of AMD usage on the dairies will be beneficial to the understanding of AMD use and the potential for AMR development on CA dairies.

## Conclusions

The current study provided a description of the current AMD use, treatments, and AMD stewardship practices that may influence AMR on CA dairies. The majority of the current practices reported by respondent CA dairy producers are in line with the provisions of the SB 27 and VFD regulations. The purpose of such regulations is to protect human and animal health through the judicious use of MIADs in food-producing animals, which has the potential to slow or prevent the development of bacterial resistance to AMDs. Support for producers in understanding and enhancing their VCPRs is likely to enhance judicious use of antimicrobial drugs. Producers and veterinarians may want to consider implementation of selective dry cow therapy, increased use of alternatives to AMDs, and adoption of electronic record-keeping as means to further implement antimicrobial stewardship.

## Supplemental Information

10.7717/peerj.11515/supp-1 Supplemental Information 1 Summary of dairy cow vaccination practices from 149 responses to questionnaire on antimicrobial drug use in adult cows on California dairies. ^a^ BVD = Bovine viral disease; ^b^ SRP = siderophore receptor and porin

10.7717/peerj.11515/supp-2 Supplemental Information 2 Summary of basis and antimicrobial choices for mastitis treatment from 149 responses to questionnaire on antimicrobial drug use in adult cows on California dairies.

10.7717/peerj.11515/supp-3 Supplemental Information 3 Summary of basis and antimicrobial choices for metritis treatment from 149 responses to questionnaire on antimicrobial drug use in adult cows on California dairies.

10.7717/peerj.11515/supp-4 Supplemental Information 4 Summary of basis and antimicrobial choices for lameness treatment from 149 responses to questionnaire on antimicrobial drug use in adult cows on California dairies.

10.7717/peerj.11515/supp-5 Supplemental Information 5 Summary of basis and antimicrobial choices for pneumonia and postoperative care from 149 responses to questionnaire on antimicrobial drug use in adult cows on California dairies. ^a^ DA = displaced abomasum; ^b^ C-Section = Cesarean section

10.7717/peerj.11515/supp-6 Supplemental Information 6 Summary of antimicrobial drug use and stewardship practices in adult cows on California dairies stratified by herd size, region, and involvement of veterinarian.

10.7717/peerj.11515/supp-7 Supplemental Information 7 Description of conventional and organic dairies typologies identified using Multiple factor analysis and Hierarchical clustering.

## Additional Information and Declarations

### Data Availability

The following information was supplied regarding data availability: While the raw data was available for peer review, this study was sponsored by the California Department of Food and Agriculture and is subject to California Food and Agriculture Code (FAC) Sections 14400 to 14408. FAC section 14407 requires that data collected be held confidential to prevent individual identification of a farm or business; as such, raw data from this study is not able to be shared.
