# Supplementary material for: 2018 Survey of antimicrobial drug use and stewardship practices in adult cows on California dairies: post-Senate Bill 27
Source: PeerJ. 2021 Jul 13;9:e11515. doi: 10.7717/peerj.11515 (PMC8284310; doi:10.7717/peerj.11515)
Supplement: Supplemental Information 1 — a BVD = Bovine viral disease; b SRP = siderophore receptor and porin [file peerj-09-11515-s001.docx]

|  |  |  |  | **95% Confidence limits** | |
| --- | --- | --- | --- | --- | --- |
| **Question** | **n** | **Estimate (%)** | **SE** | **Lower** | **Upper** |
| Mastitis vaccine [*Coliform*] |  |  |  |  |  |
| Yes | 119 | 85.6 | 2.9 | 78.6 | 90.5 |
| No | 20 | 14.3 | 2.9 | 9.4 | 21.3 |
| Mastitis Vaccine [*Staphylococcus*] |  |  |  |  |  |
| Yes | 11 | 7.7 | 2.2 | 4.3 | 13.5 |
| No | 131 | 92.2 | 2.2 | 86.4 | 95.6 |
| Diarrhea/Scours vaccine [*E. coli*, Rota, Corona] |  |  |  |  |  |
| Yes | 50 | 35.4 | 4.0 | 27.9 | 43.7 |
| No | 91 | 64.5 | 4.0 | 56.2 | 72.0 |
| Respiratory disease vaccine |  |  |  |  |  |
| Yes | 121 | 87.0 | 2.8 | 80.3 | 91.7 |
| No | 18 | 12.9 | 2.8 | 8.2 | 19.6 |
| Abortion/infertility vaccine [Leptospirosis, BVD^a^] |  |  |  |  |  |
| Yes | 103 | 73.0 | 3.7 | 65.0 | 79.7 |
| No | 38 | 26.9 | 3.7 | 20.2 | 34.9 |
| Pinkeye vaccine |  |  |  |  |  |
| Yes | 25 | 18.1 | 3.2 | 12.5 | 25.5 |
| No | 113 | 81.8 | 3.2 | 74.4 | 87.4 |
| *Clostridium* vaccine |  |  |  |  |  |
| Yes | 79 | 55.6 | 4.1 | 47.3 | 63.6 |
| No | 63 | 44.3 | 4.1 | 36.3 | 52.6 |
| Footrot vaccine |  |  |  |  |  |
| Yes | 1 | 0.7 | 0.7 | 0.01 | 4.9 |
| No | 140 | 99.2 | 0.7 | 95.0 | 99.9 |
| *Salmonella* vaccine [SRP^b^] |  |  |  |  |  |
| Yes | 7 | 4.9 | 1.8 | 2.3 | 10.1 |
| No | 134 | 95.0 | 1.8 | 89.8 | 97.6 |
