# Supplementary material for: 2018 Survey of antimicrobial drug use and stewardship practices in adult cows on California dairies: post-Senate Bill 27
Source: PeerJ. 2021 Jul 13;9:e11515. doi: 10.7717/peerj.11515 (PMC8284310; doi:10.7717/peerj.11515)
Supplement: Supplemental Information 2 [file peerj-09-11515-s002.docx]

|  |  | **Estimate (%)** |  | **95% Confidence limits** | |
| --- | --- | --- | --- | --- | --- |
| **Question** | **n** |  | **SE** | **Lower** | **Upper** |
| Mastitis: Treatment incidence per 100 milking cow months | 88 | 2.9 | 0.3 | 2.2 | 3.7 |
| Mastitis: Basis for treatment decision |  |  |  |  |  |
| Findings of abnormal milk | 48 | 36.9 | 4.2 | 29.0 | 45.6 |
| Abnormal milk + Lab testing | 33 | 25.3 | 3.8 | 18.5 | 33.6 |
| Abnormal milk + Lab testing + Treat pending test result | 49 | 37.6 | 4.2 | 29.7 | 46.3 |
| Mastitis: Choice of antimicrobial treatment |  |  |  |  |  |
| Intramammary | 99 | 76.7 | 3.7 | 68.6 | 83.2 |
| Intramammary + Oral/Injectables | 30 | 23.3 | 3.7 | 16.7 | 31.3 |
| Mastitis: First choice of drug for intramammary treatment |  |  |  |  |  |
| Cephalosporins | 105 | 85.3 | 3.1 | 77.8 | 90.6 |
| Penicillins | 9 | 7.3 | 2.3 | 3.8 | 13.5 |
| Lincosamides | 7 | 5.6 | 2.0 | 2.7 | 11.5 |
| Tetracycline | 2 | 1.6 | 1.1 | 0.4 | 6.3 |
| Mastitis: Second choice of drug for intramammary treatment |  |  |  |  |  |
| Cephalosporins | 23 | 36.5 | 6.0 | 25.4 | 49.2 |
| Lincosamides | 21 | 33.3 | 5.9 | 22.6 | 46.0 |
| Penicillins | 16 | 25.3 | 5.4 | 16.0 | 37.7 |
| Cephalosporins + Lincosamides | 2 | 3.1 | 2.2 | 0.7 | 12.1 |
| Sulfonamides | 1 | 1.5 | 1.5 | 0.2 | 10.7 |
| Mastitis: First choice of drug for oral/injectable treatment |  |  |  |  |  |
| Cephalosporins | 4 | 22.2 | 9.7 | 7.9 | 48.5 |
| Penicillins | 5 | 27.7 | 10.5 | 11.2 | 53.8 |
| Sulfonamides | 5 | 27.7 | 10.5 | 11.2 | 53.8 |
| Tetracycline | 4 | 22.2 | 9.7 | 7.9 | 48.5 |
| Mastitis: Second choice of drug for oral/injectable treatment |  |  |  |  |  |
| Penicillins | 3 | 42.8 | 18.7 | 10.3 | 82.9 |
| Sulfonamides | 2 | 28.5 | 17.0 | 4.9 | 75.6 |
| Tetracycline | 1 | 14.2 | 13.2 | 1.1 | 70.0 |
| Lincosamides | 1 | 14.2 | 13.2 | 1.1 | 70.0 |
