# Supplementary material for: 2018 Survey of antimicrobial drug use and stewardship practices in adult cows on California dairies: post-Senate Bill 27
Source: PeerJ. 2021 Jul 13;9:e11515. doi: 10.7717/peerj.11515 (PMC8284310; doi:10.7717/peerj.11515)
Supplement: Supplemental Information 3 [file peerj-09-11515-s003.docx]

Appendix 3

Summary of basis and antimicrobial choices for metritis treatment from 149 responses to questionnaire on antimicrobial drug use in adult cows on California dairies

|  |  |  |  | **95% Confidence limits** | |
| --- | --- | --- | --- | --- | --- |
| **Question** | **n** | **Estimate (%)** | **SE** | **Lower** | **Upper** |
| Metritis: Treatment incidence per 100 milking cow months | 73 | 1.9 | 0.2 | 1.3 | 2.4 |
| Metritis: Basis for treatment decision |  |  |  |  |  |
| Clinical presentation (hard calving, retained placenta, virgin discharge) | 38 | 31.9 | 4.2 | 24.1 | 40.9 |
| Clinical examination (palpation, rectal temp, treat all fresh cow) | 8 | 6.7 | 2.2 | 3.3 | 12.9 |
| Clinical presentation + Clinical examination | 73 | 61.3 | 4.4 | 52.2 | 69.7 |
| Metritis: Choice of antimicrobial treatment |  |  |  |  |  |
| Bolus/Injectables | 93 | 75.6 | 3.8 | 67.1 | 82.4 |
| Intrauterine | 9 | 7.3 | 2.3 | 3.8 | 13.5 |
| Intrauterine + Bolus/Injectables | 21 | 17.0 | 3.3 | 11.3 | 24.8 |
| Metritis: First choice of drug for Intrauterine treatment |  |  |  |  |  |
| Tetracycline | 11 | 52.3 | 10.8 | 30.6 | 73.2 |
| Cephalosporins | 6 | 28.5 | 9.8 | 12.7 | 52.2 |
| Penicillins | 4 | 19.0 | 8.5 | 6.8 | 42.8 |
| Metritis: Second choice of drug for Intrauterine treatment |  |  |  |  |  |
| Tetracycline | 3 | 42.8 | 18.7 | 10.3 | 82.9 |
| Cephalosporins | 3 | 42.8 | 18.7 | 10.3 | 82.9 |
| Penicillins | 1 | 14.2 | 13.2 | 1.1 | 70.0 |
| Metritis: First choice of drug for bolus/injectable treatment |  |  |  |  |  |
| Cephalosporins | 73 | 76.0 | 4.3 | 66.3 | 83.6 |
| Penicillins | 19 | 19.7 | 4.0 | 12.9 | 29.0 |
| Tetracycline | 3 | 3.1 | 1.7 | 0.9 | 9.3 |
| Sulfonamides | 1 | 1.0 | 1.0 | 0.1 | 7.1 |
| Metritis: Second choice of drug for bolus/injectable treatment |  |  |  |  |  |
| Penicillins | 40 | 68.9 | 6.0 | 55.7 | 79.6 |
| Cephalosporins | 17 | 29.3 | 5.9 | 18.8 | 42.4 |
| Tetracycline | 1 | 1.7 | 1.7 | 0.2 | 11.6 |
