# Supplementary material for: 2018 Survey of antimicrobial drug use and stewardship practices in adult cows on California dairies: post-Senate Bill 27
Source: PeerJ. 2021 Jul 13;9:e11515. doi: 10.7717/peerj.11515 (PMC8284310; doi:10.7717/peerj.11515)
Supplement: Supplemental Information 4 [file peerj-09-11515-s004.docx]

|  |  | **Estimate (%)** |  | **95% Confidence limits** | |
| --- | --- | --- | --- | --- | --- |
| **Question** | **n** |  | **SE** | **Lower** | **Upper** |
| Lameness: Treatment incidence per 100 milking cow months | 79 | 1.5 | 0.2 | 0.9 | 2.0 |
| Lameness: Basis for treatment decision |  |  |  |  |  |
| Rely on lameness signs | 34 | 26.5 | 3.9 | 19.5 | 34.9 |
| Hoof trimmer exam | 27 | 21.0 | 3.6 | 14.8 | 29.0 |
| Hoof trimmer exam + Rely on lameness signs | 67 | 52.3 | 4.4 | 43.6 | 60.9 |
| Lameness: Choice of antimicrobial treatment |  |  |  |  |  |
| Hoof treatment (antibiotic wrap, heel spray, foot bath) | 33 | 28.9 | 4.2 | 21.2 | 38.0 |
| Bolus/Injectables | 16 | 14.0 | 3.2 | 8.7 | 21.7 |
| Hoof treatment + Bolus/Injectables | 65 | 57.0 | 4.6 | 47.6 | 65.8 |
| Lameness: First choice of drug for hoof treatment |  |  |  |  |  |
| Tetracycline | 32 | 68.0 | 6.7 | 53.1 | 80.0 |
| Cephalosporins | 11 | 23.4 | 6.1 | 13.2 | 37.9 |
| Lincosamides | 2 | 4.2 | 2.9 | 1.0 | 15.9 |
| Sulfonamides | 2 | 4.2 | 2.9 | 1.0 | 15.9 |
| Lameness: Second choice of drug for hoof treatment |  |  |  |  |  |
| Tetracycline | 5 | 38.4 | 13.4 | 15.2 | 68.3 |
| Penicillins | 3 | 23.0 | 11.6 | 6.6 | 55.7 |
| Cephalosporins | 2 | 15.3 | 10.0 | 3.2 | 49.2 |
| Lincosamides | 2 | 15.3 | 10.0 | 3.2 | 49.2 |
| Sulfonamides | 1 | 7.6 | 7.3 | 0.8 | 44.5 |
| Lameness: First choice of drug for bolus/injectable treatment |  |  |  |  |  |
| Cephalosporins | 40 | 54.0 | 5.7 | 42.4 | 65.1 |
| Sulfonamides | 18 | 24.3 | 4.9 | 15.7 | 35.5 |
| Penicillins | 15 | 20.2 | 4.6 | 12.5 | 31.1 |
| Macrolides | 1 | 1.3 | 1.3 | 0.1 | 9.2 |
| Lameness: Second choice of drug for injectable treatment |  |  |  |  |  |
| Penicillins | 12 | 52.1 | 10.4 | 31.4 | 72.1 |
| Sulfonamides | 7 | 30.4 | 9.5 | 14.5 | 52.8 |
| Cephalosporins | 3 | 13.0 | 7.0 | 3.9 | 35.1 |
| Tetracycline | 1 | 4.3 | 4.2 | 0.5 | 27.4 |
