# Supplementary material for: 2018 Survey of antimicrobial drug use and stewardship practices in adult cows on California dairies: post-Senate Bill 27
Source: PeerJ. 2021 Jul 13;9:e11515. doi: 10.7717/peerj.11515 (PMC8284310; doi:10.7717/peerj.11515)
Supplement: Supplemental Information 5 — a DA = displaced abomasum; b C-Section = Cesarean section [file peerj-09-11515-s005.docx]

|  |  | **Estimate (%)** |  | **95% Confidence limits** | |
| --- | --- | --- | --- | --- | --- |
| **Question** | **n** |  | **SE** | **Lower** | **Upper** |
| Pneumonia: Treatment incidence per 100 milking cow months | 66 | 0.3 | 0.1 | 0.2 | 0.5 |
| Pneumonia: Basis for treatment decision |  |  |  |  |  |
| Respiratory clinical signs (cough, difficult breathing, nasal disc) | 112 | 97.3 | 1.4 | 92.1 | 99.1 |
| Not rely on respiratory clinical signs | 3 | 2.6 | 1.4 | 0.8 | 7.8 |
| Pneumonia: Choice of antimicrobial treatment |  |  |  |  |  |
| Injectables/bolus: Yes | 109 | 98.1 | 1.2 | 92.9 | 99.5 |
| Injectables/bolus: No | 2 | 1.8 | 1.2 | 0.4 | 7.0 |
| Pneumonia: First choice of drug for injectable treatment |  |  |  |  |  |
| Cephalosporins | 59 | 57.8 | 4.8 | 47.9 | 67.1 |
| Penicillins | 21 | 20.5 | 4.0 | 13.7 | 29.6 |
| Tetracycline | 8 | 7.8 | 2.6 | 3.9 | 15.0 |
| Amphenicols | 6 | 5.8 | 2.3 | 2.6 | 12.5 |
| Sulfonamides | 5 | 4.9 | 2.1 | 2.0 | 11.3 |
| Fluoroquinolones | 2 | 1.9 | 1.3 | 0.4 | 7.6 |
| Macrolides | 1 | 0.9 | 0.9 | 0.1 | 6.7 |
| Pneumonia: Second choice of drug for injectable treatment |  |  |  |  |  |
| Penicillins | 15 | 42.8 | 8.3 | 27.2 | 60.0 |
| Cephalosporins | 11 | 31.4 | 7.8 | 17.9 | 48.9 |
| Tetracycline | 4 | 11.4 | 5.3 | 4.1 | 27.5 |
| Sulfonamides | 2 | 5.7 | 3.9 | 1.3 | 21.0 |
| Amphenicols | 1 | 2.8 | 2.8 | 0.3 | 18.7 |
| Fluoroquinolones | 1 | 2.8 | 2.8 | 0.3 | 18.7 |
| Macrolides | 1 | 2.8 | 2.8 | 0.3 | 18.7 |
|  |  |  |  |  |  |
| Post-surgery: Treatment incidence per 100 milking cow months | 65 | 0.04 | 0.01 | 0.01 | 0.06 |
| Post-surgery: Basis for treatment decision |  |  |  |  |  |
| Routinely after DA^a^ or C-Section^b^ | 20 | 27.3 | 5.2 | 18.2 | 38.9 |
| Rely on veterinarian instruction | 36 | 49.3 | 5.8 | 37.8 | 60.8 |
| Routinely after DA + rely on veterinarian | 17 | 23.2 | 4.9 | 14.8 | 34.5 |
| Post-surgery: Choice of antimicrobial treatment |  |  |  |  |  |
| Injectables/bolus: Yes | 62 | 96.8 | 2.1 | 88.0 | 99.2 |
| Injectables/bolus: No | 2 | 3.1 | 2.1 | 0.7 | 11.9 |
| Post-surgery: First choice of drug for injectable treatment |  |  |  |  |  |
| Penicillins | 42 | 72.4 | 5.8 | 59.3 | 82.5 |
| Cephalosporins | 15 | 25.8 | 5.7 | 16.0 | 38.8 |
| Tetracycline | 1 | 1.7 | 1.7 | 0.2 | 11.6 |
| Post-surgery: Second choice of drug for injectable treatment |  |  |  |  |  |
| Penicillins | 12 | 57.1 | 10.7 | 34.7 | 76.9 |
| Cephalosporins | 6 | 28.5 | 9.8 | 12.7 | 52.2 |
| Tetracycline | 3 | 14.2 | 7.6 | 4.3 | 37.9 |
