# Supplementary material for: 2018 Survey of antimicrobial drug use and stewardship practices in adult cows on California dairies: post-Senate Bill 27
Source: PeerJ. 2021 Jul 13;9:e11515. doi: 10.7717/peerj.11515 (PMC8284310; doi:10.7717/peerj.11515)
Supplement: Supplemental Information 7 [file peerj-09-11515-s007.docx]

| **Dairy** |  | **Conventional (%)** | | **Organic (%)** |
| --- | --- | --- | --- | --- |
| **Components** | **Characteristics** | **Cluster C1** | **Cluster C2** |  |
|  |  | **N =122** | **N = 10** | **N = 16** |
| Herd demography | Region |  |  |  |
|  | NCA^c^ | 6.6 | 0.0 | 81.2 |
|  | NSJV^d^ | 43.4 | 40.0 | 0.0 |
|  | GSCA^e^ | 50.0 | 60.0 | 18.8 |
|  | Breed |  |  |  |
|  | Other | 26.4 | 30.0 | 50.0 |
|  | Hostein | 69.4 | 70.0 | 37.5 |
|  | Jersey | 4.1 | 0.0 | 12.5 |
|  | Population |  |  |  |
|  | Herd size (median) | 1,265.0 | 715.0 | 325.0 |
|  | Rolling herd average (median) | 11,339.8 | 10,092.4 | 8,235.8 |
| Dry-off protocol | Blanket^f^ treatment of all dry cow |  |  |  |
|  | No | 4.9 | 20.0 | 68.8 |
|  | Yes | 95.1 | 80.0 | 31.2 |
|  | Antimicrobial used in dry cow treatment |  |  |  |
|  | Cephalosporins | 50.9 | 66.7 | 0.0 |
|  | Penicillins | 27.1 | 0.0 | 100.0 |
|  | Cephalosporins or Penicillins | 10.5 | 0.0 | 0.0 |
|  | Penicillins or Aminoglycosides | 11.4 | 33.3 | 0.0 |
| Disease management | Mastitis: Basis for treatment decision |  |  |  |
|  | Findings of abnormal milk | 34.5 | 100.0 | 40.0 |
|  | Abnormal milk + Lab testing | 25.2 | 0.0 | 40.0 |
|  | Abnormal milk + Lab testing + Treat pending test result | 40.3 | 0.0 | 20.0 |
|  | Mastitis: Treat with antimicrobial |  |  |  |
|  | No | 1.6 | 25.0 | 81.2 |
|  | Yes | 98.4 | 75.0 | 18.8 |
|  | Metritis: Choice of antimicrobial treatment |  |  |  |
|  | Bolus/Injectables | 75.2 | 100.0 | 50.0 |
|  | Intrauterine | 6.8 | 0.0 | 50.0 |
|  | Intrauterine + Bolus/Injectables | 17.9 | 0.0 | 0.0 |
|  | Lameness: Choice of antimicrobial treatment |  |  |  |
|  | Hoof treatment (antibiotic wrap, heel spray, foot bath) | 28.4 | 66.7 | 0.0 |
|  | Bolus/Injectables | 14.7 | 0.0 | 33.3 |
|  | Hoof treatment + Bolus/Injectables | 56.8 | 33.3 | 66.7 |
| Antimicrobial stewardship | How do you track antimicrobial treatments given to cows? |  |  |  |
|  | Computer + Others^g^ | 69.7 | 40.0 | 41.7 |
|  | No computer | 30.3 | 60.0 | 58.3 |
|  | How you track antimicrobial withdrawal period for treated cows |  |  |  |
|  | Computer + Others^g^ | 66.4 | 22.2 | 37.5 |
|  | No computer | 33.6 | 77.8 | 62.5 |
|  | Administration of appropriate antimicrobial drug, dose, route, duration |  |  |  |
|  | Very important | 97.5 | 80.0 | 76.9 |
|  | Some importance | 2.5 | 20.0 | 15.3 |
|  | Not important | 0.0 | 0.0 | 7.7 |
|  | Good record keeping on treatment and treatment dates |  |  |  |
|  | Very important | 96.7 | 85.7 | 76.9 |
|  | Some importance | 3.3 | 14.3 | 15.4 |
|  | Not important | 0.0 | 0.0 | 7.7 |
|  | Observing withdrawal periods and drug residue avoidance |  |  |  |
|  | Very important | 99.2 | 100.0 | 76.9 |
|  | Some importance | 0.8 | 0.0 | 7.7 |
|  | Not important | 0.0 | 0.0 | 15.4 |
| Antimicrobial use on dairies | Used of OTC^h^ and prescription antimicrobial on dairy before 1.1.2018 |  |  |  |
|  | Both OTC and prescription AMD were used to treat cows | 69.4 | 25.0 | 53.3 |
|  | Cows were only treated with prescription AMD | 16.5 | 25.0 | 6.7 |
|  | Cows were only treated with OTC AMD | 1.7 | 25.0 | 6.7 |
|  | Cows were not treated with OTC AMD | 10.7 | 0.0 | 13.3 |
|  | Cows were not treated with prescription AMD | 0.8 | 25.0 | 0.0 |
|  | Cows were neither treated with OTC nor prescription AMD | 0.8 | 0.0 | 20.0 |
|  | Used of OTC and prescription AMD on dairy before 1.1.2018 (dichotomous response) |  |  |  |
|  | Cows were treated with OTC AMD | 72.3 | 66.7 | 75.0 |
|  | Cows were not treated with OTC AMD | 27.7 | 33.3 | 25.0 |
| Producer perceptions of AMD on dairies | Any use of antimicrobial may result in infections more difficult to treat in future |  |  |  |
|  | Strongly agree / Agree | 11.6 | 40.0 | 38.5 |
|  | Neutral | 30.6 | 20.0 | 23.0 |
|  | Strongly disagree / Disagree | 57.9 | 40.0 | 38.5 |
